# Surface Tension Isotherms: Reconceptualizing Adsorption, Self-Assembly, and Micelle Formation via the Fluctuation Theory

**DOI:** 10.1021/acs.langmuir.5c05155

**Published:** 2026-02-09

**Authors:** Seishi Shimizu, Nobuyuki Matubayasi

**Affiliations:** † York Structural Biology Laboratory, Department of Chemistry, 8748 University of York, Heslington, York YO10 5DD, United Kingdom; ‡ Division of Chemical Engineering, Graduate School of Engineering Science, 13013 Osaka University, Toyonaka, Osaka 560-8531, Japan

## Abstract

Given a surface tension isotherm (i.e., how interfacial free energy changes with the surfactant concentration), can we gain insight into how surfactant molecules interact at the interface and in the bulk solution? Historically, surfactants were modeled to bind onto a uniform interface, before aggregating stoichiometrically at the critical micelle concentration (CMC). However, this simple model contrasts with counterevidence, e.g., premicelles (smaller aggregates below CMC) and aggregate size distribution, necessitating a departure from stoichiometric aggregation models. To this end, a novel theory for surface tension will be established by synthesizing the statistical thermodynamic fluctuation theory for sorption and self-assembly in solution. This theory provides a link between the functional shape of a surface tension isotherm and the underlying interactions. We demonstrate that the gradient and curvature of a surface tension isotherm reveal a competition between surfactant sorption and bulk number fluctuation without employing any model assumptions. This novel theory proposes to (i) redefine the surfactant aggregation number using number fluctuations to replace the stoichiometric model, (ii) generalize the Szyszkowski–Langmuir isotherm (which implicitly assumes site-specific adsorption on uniformly distributed binding sites) with the novel ABC isotherm to capture the surface–bulk difference of surfactant number correlation, and (iii) replace the surfactant “area-per-molecule” with the projected area, by incorporating the thickness of the interface. This theory can be applied to surfactants and small molecules (e.g., alcohols) alike, eliminating the need for separate models over a spectrum of self-association propensities.

## Graphical abstract

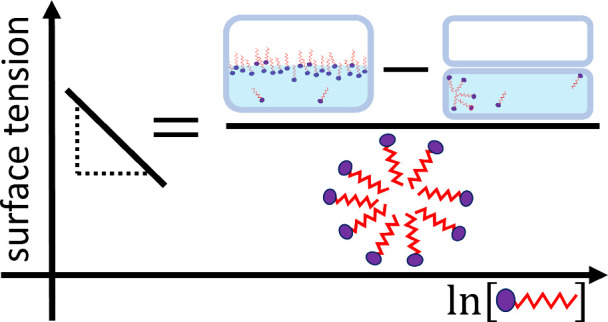

## Introduction

The surface tension of the air/water interface changes with the concentration of the surfactant (“surface tension isotherm”).^1–3^ How can we understand the functional shape of a surface tension isotherm (Figure 1) on a mechanistic basis, in terms of the underlying surfactant interactions at the interface and in the bulk solution? To answer this question with clarity, this paper proposes to replace the classical models of surface tension (based on stoichiometric aggregation and binding)^4–7^ with the statistical thermodynamic fluctuation theory.^8,9^ This proposal has been necessitated by the mounting evidence for non-stoichiometric behavior in (1) self-aggregation in the bulk (e.g., micelle formation) and (2) adsorption at air/water interface, which poses difficulties for the stoichiometric models for binding and association, as well as by (3) the interpretive difficulties caused by the oversimplifications in capturing 1 and 2, as will be summarized below.

**Figure 1:**
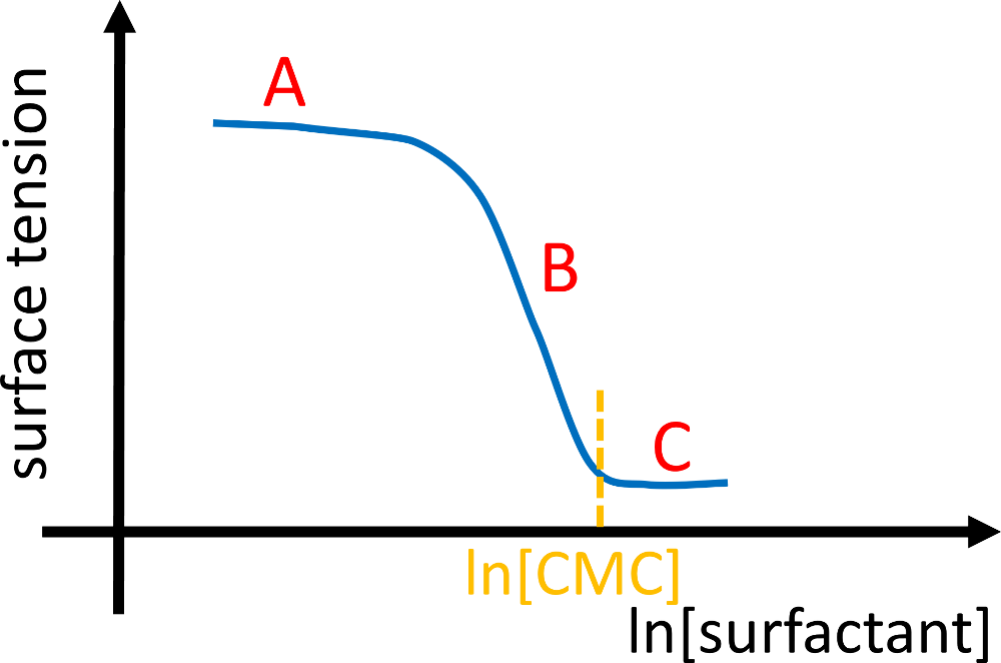
Schematic representation of a surface tension isotherm of surfactant–water mixture against the logarithmic surfactant concentration, with the regions defined by Menger et al.^10^ In region A, “the surface tension remain(s) relatively constant”.^10^ In region B, or the linear region (concentrations below CMC), “the slope of the curve is essentially constant”.^10^ In region C, above the critical micelle concentration, the surface tension levels off.^10^

### Capturing Self-Assembly in the Bulk Solution

#### Surfactants

Surfactant self-assembly cannot be captured by the stoichiometric *m*-mer formation^4,5^ that occurs abruptly at the critical micelle concentration (CMC). Evidence abounds that is contrary to the stoichiometric model: (i) premicelles (smaller surfactant aggregates) below CMC^11–13^ and (ii) micellar size distribution,^4,5,14,15^ namely, the statistical distribution of the aggregation number. Consequently, the surfactant self-assembly must be captured with appreciation of the variability and fluctuation of the aggregation number.

#### Different Aggregation Theories for Surfactants and Non-surfactants

Common experimental techniques (e.g., small-angle scattering) have been established to quantify aggregation behaviors across varying degrees of self-association, such as co-solvents and hydrotropes^16–19^ as well as surfactants^20–23^ in water. However, different theories and models are used for these three classes. For co-solvents and hydrotropes,^24–27^ their weak, non-specific, and non-stoichiometric aggregation can be captured^28–31^ through the Kirkwood–Buff integral (KBI),^32^ defined via radial distribution functions between molecular species, founded on statistical thermodynamic fluctuation theory.^33–36^ For surfactants, in contrast, aggregation numbers, founded on stoichiometric models, are still the common language.^1–7^ Despite long-standing attempts to introduce the fluctuation theory for micelles,^37–40^ stoichiometric binding models are still commonly used to capture surfactant aggregation, especially in the context of surface tension.^6,7,41^ Therefore, following previous attempts,^37–40^ our goal is to establish the fluctuation theory as the common theoretical foundation across the spectrum of self-association, applicable to co-solvents, hydrotropes, and surfactants alike.

### Capturing Adsorption on Air/Water Interface

#### What Constitutes a Full Surface Coverage?

To understand a surface tension isotherm mechanistically, not only surfactant aggregation in the bulk, but also surfactant adsorption onto the interface, must be considered.^1–3^ To this end, the Gibbs adsorption isotherm plays a crucial role, which relates the gradient of a surface tension isotherm (plotted against the logarithmic surfactant concentration) to the adsorption capacity of surfactants^42^ (i.e., the number of surfactants adsorbed per area). Therefore, the commonly observed linear region in the surface tension isotherm (Figure 1) can be attributed to the adsorption capacity reaching saturation.^1–3^ To understand more microscopically the surface coverage of surfactants at saturation, the area-per-molecule is a useful measure to obtain a microscopic picture of surface coverage, easily calculable as the inverse of the saturation capacity (“the number of surfactants per area”).^1^ However, the area-per-surfactant data, collected extensively (see, e.g., Table 2.1 by Rosen and Kunjappu^1^) in the literature, have recently been questioned by Menger and co-workers.^10,43,44^ The area values seemed too large [“[i]­n order to achieve large surface tension reductions (typically from 72 mN/m to 30–40 mN/m), the interface must be packed far more tightly”^10^] and “surprisingly insensitive to rather sizable structural changes”.^44^ This has escalated to the controversy on the foundation of the Gibbs isotherm.^10,43–47^

#### Molecular Basis of Surface Coverage

How can we judge whether the area-per-surfactant is too large? To answer this question, the adsorption capacity (i.e., the inverse of area-per-surfactant) must be elucidated microscopically. However, the common isotherm models are incapable of answering this important question. The Szyzykowski–Langmuir model is the most common one for surface tension, which (despite its genesis as an empirical model^48,49^ ) represents^50^ surfactant adsorption onto identical binding sites distributed uniformly on a homogeneous surface.^8,51^ However, mounting experimental evidence shows “the existence of thick adsorption layers, especially for ionic surfactants”.^52^ which is in contrast with “adsorption layers without thickness” that “are assumed by most adsorption models, resulting in an infinite concentration of the surfactant at the air–water interface, which is not supported by experimental results”.^52^ This poses serious questions on the meaning of (i) the number of binding sites as a parameter in the Szyszkowski–Langmuir model, with no further explanation to be gained about their physical origin, and (ii) the meaning of the “area-per-molecule”. These limitations have only recently been overcome by a generalization of the Langmuir model with the fluctuation theory,^8,9^ replacing the stoichiometric binding model for adsorption with the sorbate–surface and sorbate–sorbate interactions at the interface, with the allowance for interfacial thickness.^53^ This novel approach to sorption isotherms is founded on number correlations and KBIs,^54^ in the common language of the fluctuation theory shared with aggregation phenomena in the bulk solution,^55,56^ giving rise to a common theoretical foundation for sorption from gases and liquid solutions.^9^ Without a need for simple model assumptions (e.g., lattice model^57^), our approach can capture specific and non-specific interactions alike.^58^ Thus, our goal is to establish a theory for surface tension isotherms with the interactions in the bulk and at the interface formulated consistently via the fluctuation theory.

### Interpretive Difficulties of Surface Tension Isotherms

#### Surfactant Surface Tension Isotherms

The disagreements documented in the literature^1,10^ over the molecular interactions underlying the following regions of the surface tension isotherm, regarding

(A) the onset in the decline of surface tension (region A of Figure 1)
(B) the near-linear decrease in surface tension (region B of Figure 1)
(C) the plateau of the surface tension isotherm above CMC (region C of Figure 1)

are summarized in detail in Supporting Information: A. The lack of consensus in the literature necessitates a shift away from the classical way of thinking based on stoichiometric binding and aggregation models.

#### CMC-Like Behavior in Alcohol–Water Mixture

The *n*-propanol–water mixture is not considered a surfactant solution but exhibits a CMC-like behavior (Figure 2a).^59^ The need to rationalize this puzzling behavior is yet another important reason to modernize the surface tension isotherm theory. The clue to solving this paradox is again on the concentration scale: when plotted using the activity of *n*-propanol, the CMC-like behavior disappears; the surface tension isotherm of the *n*-propanol–water mixture becomes virtually identical to that of the ethanol–water mixture (Figure 2b).^59^ However, what precisely causes the CMC-like behavior when plotted against the mole fraction instead of activity? Can we establish a common theory for alcohols and surfactants? These questions will be addressed by statistical thermodynamic fluctuation theory, which has a proven track record of providing mechanistic insights into solvation^58^ and adsorption,^8,9,60^ regardless of the molecular scales and aggregation tendencies.

**Figure 2:**
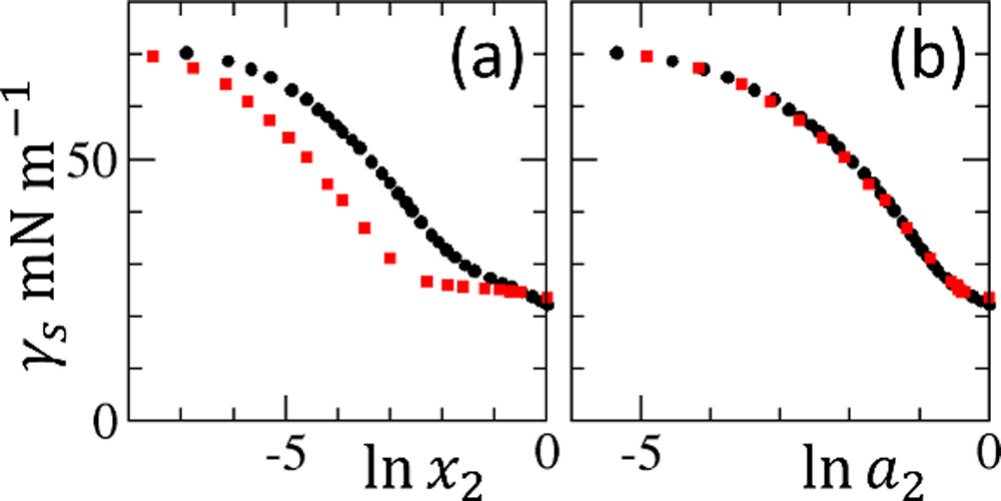
Surface tension isotherms of aqueous ethanol (black circles) and *n*-propanol (red squares) solutions, denoted with 1 = water and 2 = alcohol, (a) against ln *x*_2_ and (b) against ln *a*_2_ using the published data by Strey et al. at 25 °C.^59^ Note that the difference between ethanol and *n*-propanol becomes apparent in the ln *x*_2_ plot a, with the apparent CMC-like behavior for *n*-propanol (i.e., a near-zero gradient at ln *x*_2_ > −2.5) that is not observed in plot b, the ln *a*_2_ plot.^59^

### Aims and Objectives

Our goal is to reveal the molecular interactions underlying the functional shape of an experimental surface tension isotherm. To this end, we need a novel theory synthesizing bulk self-aggregation and surface adsorption. To encompass the diverse classes of molecules added to water,^58^ we introduce “co-molecule” as a general terminology for co-solvents, hydrotropes, and surfactants employed in the following different contexts:

- self-aggregation propensity in the bulk
- adsorption onto air–water interface as adsorbates
- surface tension modifier

In addition, the present limitations arising from the stoichiometric assumptions must be overcome. For all these classes and contexts of co-molecules, a common theoretical language must be established. To this end, our aim is to construct a universal theory of surface tension isotherms. This can be achieved by the following theoretical tools, all founded consistently on the statistical thermodynamic fluctuation theory:

(A) sorption on air–water interface (via the generalization of our recent work on solid/liquid and solid/gas isotherms^8,9,61,62^ )
(B) aggregation in bulk solution (founded on our recent work on complex solutions^31,40^ )
(C) surface tension isotherm, i.e., how the competition between A and B synthetically gives rise to its gradient (first derivative) and curvature (second derivative)
(D) how equations for surface tension isotherm can be derived via A–C.

These four theoretical tools will be crucial for achieving our fourfold objectives:

(I) to reconceptualize the surfactant aggregation number based on the fluctuation theory, clarifying how it manifests in surface tension and activity measurements
(II) to establish a common language for surface tension, adsorption, and aggregation common to all co-molecules, valid for surfactants and non-surfactants alike
(III) to describe surface excess (surface tension decrease) and surface deficit (surface tension increase) for all co-molecules in a consistent language, including the factors influencing the apparent “area-per-molecule” ^1,10,43,44^ at the interface
(IV) to provide a mechanistic explanation of every salient feature of a typical surface tension isotherm (Figure 1) without employing any model assumptions

## Theory

### Capturing Molecular Interactions at the Interface and Bulk

#### Interfacial and Bulk Subsystems

Our goal is to understand how the surface tension, γ_s_, changes with solution composition (“surface tension isotherm”) from the underlying molecular interactions. To this end, let us set up a gas–liquid interface of a binary mixture of solvent (species 1) and co-molecule (species 2) with the area of the interface, σ (note that the term “co-molecule” had to be chosen due to the need to include a wide variety of molecules for species 2; see Aims and Objectives in the Introduction section). We have shown previously that the classical foundation of interfacial thermodynamics, based on the combination of the Gibbs adsorption isotherm with the Gibbs dividing surface,^42^ can be generalized to any interfacial geometry by introducing a trio of partially open ensembles, open only to co-molecules, with the number of solvents constrained.^54^ Incorporating the local nature of the interface (i.e., the structural deviation of water–co-molecule mixtures from the bulk liquid and air, which is confined within a finite distance range from the interface,^54^) leads to the trio of partially open subsystems

- the interfacial subsystem: $\left\{T,v^{*},n_{1}^{*},\mu_{2}\right\}$

- the reference bulk liquid subsystem: $\left\{T,v^{l},n_{1}^{l},\mu_{2}\right\}$

- the reference bulk air subsystem: $\left\{T,v^{a},n_{1}^{a},\mu_{2}\right\}$

where *T* is the temperature and μ_2_ is the chemical potential of the co-molecule. We emphasize that all the intra- and intermolecular interactions (e.g., van der Waals and electrostatic) have been incorporated in the partition functions. The volume of the interfacial subsystem, v*, covers the region in which co-molecule distribution deviates from the bulk liquid and air (which may therefore be larger than the dense first layer of co-molecules), with the volume constraint

1 $$v^{*}=v^{l}+v^{a}$$

The interface is positioned such that the excess number of solvent molecules is zero, i.e.

2 $$n_{1}^{*}=n_{1}^{l}+n_{1}^{a}$$

which acts as the constraint on the number of solvents for the three subsystems involved. In addition, the bulk solvent concentrations in the air and liquid sides

3 $$c_{1}^{a}=\frac{n_{1}^{a}}{v^{a}},,c_{1}^{l}=\frac{n_{1}^{l}}{v^{l}}$$

will be crucial for specifying the sizes of the two reference subsystems. Working with the trio of partially open subsystems enables us to focus on (i) surface–co-molecule and (ii) co-molecule–co-molecule interactions, both mediated by the solvent molecules surrounding them. Consequently, the natural language for quantifying (i) and (ii) is the molecular distribution function.^54^

#### Surface–Co-molecule Interaction

Our objective here is to capture the surface–co-molecule interaction. Microscopically speaking, the distribution function $c_{2}^{*}\left(\mathrm{r⃗}\right)$ , at the position r⃗ , represents the local concentration of co-molecule. Then, the overall interfacial effect is quantified by the net excess of $c_{2}^{*}\left(\mathrm{r⃗}\right)$ from the reference bulk subsystems on the air $\left(c_{2}^{a}\right)$ and liquid $\left(c_{2}^{l}\right)$ sides (Supporting Information: B). However, it is challenging to have precise information on the functional form of $c_{2}^{*}\left(\mathrm{r⃗}\right)$ . Indeed, macroscopic observables (such as surface tension or adsorption) are determined, under the dividing condition, via the surface excess Γ_s2_, defined by

4 $$\Gamma_{s2}=\frac{\left\langle n_{2}^{*}\right\rangle -\left\langle n_{2}^{l}\right\rangle -\left\langle n_{2}^{a}\right\rangle }{\sigma }$$

where σ is the area of the interface. We will demonstrate below that mechanistic insights can be obtained from the derivatives of Γ_s2_ (eq 4), which defines the surface excess as the deviation of the mean ⟨ ⟩ number of co-molecules in the system $\left\langle n_{2}^{*}\right\rangle$ from those of the reference subsystems, $\left\langle n_{2}^{l}\right\rangle$ , and $\left\langle n_{2}^{a}\right\rangle$ (see Supporting Information: B for details).

#### Co-molecule–Co-molecule Interaction

The co-molecule–co-molecule interaction in all three subsystems (interfacial, bulk liquid, and bulk air) can be quantified by the excess number, *N*_22_, defined by

5 $$N_{22}={\left\langle n_{2}\right\rangle }_{2}-\left\langle n_{2}\right\rangle$$

where ${\left\langle n_{2}\right\rangle }_{2}$ is the number of co-molecules present in the vicinity of a probe co-molecule and ⟨*n*_2_⟩ is the number of co-molecules in the absence of a probe co-molecule, both observed in the same volume.^55,56^ A positive *N*_22_ indicates the excess of co-molecules located around the probe, while a negative *N*_22_ denotes the deficit thereof. Thus, *N*_22_ serves as an overall measure of co-molecule–co-molecule attraction or repulsion. *N*_22_ can also be expressed in terms of the mean number deviation of co-molecules, ⟨δ*n*_2_δ*n*_2_⟩, commonly referred to as the number fluctuation, via

6 $$N_{22}+1=\frac{\left\langle \delta n_{2}\delta n_{2}\right\rangle }{\left\langle n_{2}\right\rangle }$$

which is an important relationship linking co-molecule interaction to its number fluctuation. Both representations of the excess numbers (eqs 5 and 6) offer complementary perspectives to the co-molecule–co-molecule interaction, and will be employed throughout this paper.

In the next four subsections, we will develop tools A–D (see Aims and Objectives in the Introduction) based on the foundation presented above.

### Adsorption Contribution to the Surface Tension Isotherm (Tool A)

#### Surface–Co-molecule Interaction

The first derivative (gradient) of a surface tension isotherm (γ_s_) reveals the underlying surface–co-molecule interaction, captured by the surface excess Γ_s2_, through (Supporting Information: B)­

7 $$-\frac{1}{RT}{\left(\frac{\partial \gamma_{s}}{\partial \ln a_{2}}\right)}_{T}=\Gamma_{s2}$$

where *a*_2_ is the activity of the co-molecule and *R* is the gas constant.^53,54^ We emphasize here that our generalization of the Gibbs isotherm (eq 7) is an exact relationship founded on the partially open ensembles (eq 2) for the generalized dividing condition.^53,54^

#### Co-molecule–Co-molecule Interaction

The second derivative (curvature) of the surface tension isotherm reveals the underlying co-molecule–co-molecule interactions via (see Supporting Information: B)­

8 $$\begin{array}{l} -\frac{1}{RT}{\left(\frac{\partial^{2}\gamma_{s}}{\partial {\left[\ln a_{2}\right]}^{2}}\right)}_{T}={\left(\frac{\partial \Gamma_{s2}}{\partial \ln a_{2}}\right)}_{T}=\frac{\Delta_{s}\left\langle \delta n_{2}\delta n_{2}\right\rangle }{\sigma }\\ \Delta_{s}\left\langle \delta n_{2}\delta n_{2}\right\rangle \equiv \left\langle \delta n_{2}^{*}\delta n_{2}^{*}\right\rangle -\left\langle \delta n_{2}^{l}\delta n_{2}^{l}\right\rangle -\left\langle \delta n_{2}^{a}\delta n_{2}^{a}\right\rangle \end{array}$$

where $\left\langle \delta n_{2}^{*}\delta n_{2}^{*}\right\rangle$ , $\left\langle \delta n_{2}^{l}\delta n_{2}^{l}\right\rangle$ , and $\left\langle \delta n_{2}^{a}\delta n_{2}^{a}\right\rangle$ are the co-molecule number fluctuations in the subsystems at the interface, reference l, and reference a, respectively.^63^ Here, we express Δ_s_⟨δ*n*_2_δ*n*_2_⟩ in eq 8, referred to as the surface excess fluctuation,^63^ in terms of the excess numbers (eq 6), as

9 $$\Delta_{s}\left\langle \delta n_{2}\delta n_{2}\right\rangle =\left\langle n_{2}^{*}\right\rangle \left(N_{22}^{*}+1\right)-\left\langle n_{2}^{l}\right\rangle \left(N_{22}^{l}+1\right)-\left\langle n_{2}^{a}\right\rangle \left(N_{22}^{a}+1\right)$$

This excess number representation (eq 9) provides a seamless link between adsorption and bulk aggregation of co-molecules (tool B) and will be central to deriving isotherm equations (tool C).

### Bulk Self-Assembly Contribution to the Surface Tension Isotherm (Tool B)

#### Need for Consistency with the Adsorption Theory

To clarify how self-assembly in the bulk solution contributes to the surface tension isotherm (tool C), the theory of self-assembly must be formulated in a manner consistent with the adsorption theory (tool A). Crucial for achieving this end will be the relationship between co-molecule activity and concentration, which appears in surface tension and bulk solution measurements (for co-molecule concentration, we will focus chiefly on molality, defined via $m_{2}=n_{2}^{l}/\left(M_{1}n_{1}^{l}\right)$ , with *M*_1_ as the molecular weight of the solvent, due to its direct link to co-molecule self-assembly in the bulk, as will be shown in the next subsection). Each point in a surface tension isotherm is a measurement of surface tension for a solution with a concentration of a co-molecule $m_{2}^{\prime }$ prepared under pressure *P*′. Since the chemical potential $\mu_{2}^{\prime }$ of this bulk solution is the same as the one in the interfacial system $\mu_{2}=\mu_{2}^{\prime }$ , how μ_2_ changes with $m_{2}^{\prime }$ can be expressed via

10 $$d\mu_{2}={\left(\frac{\partial \mu_{2}^{\prime }}{\partial P^{\prime }}\right)}_{T,m_{2}^{\prime }}dP^{\prime }+{\left(\frac{\partial \mu_{2}^{\prime }}{\partial m_{2}^{\prime }}\right)}_{T,P^{\prime }}dm_{2}^{\prime }$$

Since $v_{2}^{\prime }={\left(\frac{\partial \mu_{2}^{\prime }}{\partial P^{\prime }}\right)}_{T,m_{2}^{\prime }}$ is the partial molar volume of co-molecule in the bulk solution and the molality is common between the interfacial and bulk solution, i.e., $m_{2}^{\prime }=m_{2}$ , eq 10 can be differentiated for the interfacial system to yield

11 $$\begin{array}{lll} {\left(\frac{\partial \mu_{2}}{\partial m_{2}}\right)}_{T,\mathrm{interface}} & = & v_{2}^{\prime }{\left(\frac{\partial P^{\prime }}{\partial m_{2}}\right)}_{T;\mathrm{interface}}+{\left(\frac{\partial \mu_{2}}{\partial m_{2}}\right)}_{T,P;\mathrm{bulk}}\\ & ≃ & {\left(\frac{\partial \mu_{2}}{\partial m_{2}}\right)}_{T,P;\mathrm{bulk}} \end{array}$$

where the negligibility of $v_{2}^{\prime }{\left(\frac{\partial P^{\prime }}{\partial m_{2}}\right)}_{T;\mathrm{interface}}$ in eq 11 can be justified via a simple order-of-magnitude analysis (see Supporting Information: C). Thus, we can employ activity measurements for bulk solution systems to interpret surface tension isotherms reported against co-molecule concentrations. Its especially useful consequence is $N_{22}^{l}$ for the liquid reference (that appears also in eq 9), which can now be obtained from the bulk measurements of the chemical potential μ_2_ against the molality *m*_2_ (see Supporting Information: C), via

12 $$\frac{1}{RT}{\left(\frac{\partial \ln m_{2}}{\partial \mu_{2}}\right)}_{T,P;\mathrm{bulk}}=N_{22}^{l}+1$$

From eq 12, we obtain a relationship for converting *m*_2_ to ln *a*_2_

13 $${\left(\frac{\partial \ln a_{2}}{\partial \ln m_{2}}\right)}_{T;\mathrm{interface}}=\frac{1}{N_{22}^{l}+1}$$

and for converting the mole-fraction scale (*x*_2_ = *n*_2_/(*n*_1_ + *n*_2_)) to ln *x*_2_

14 $${\left(\frac{\partial \ln a_{2}}{\partial \ln x_{2}}\right)}_{T,P,n_{1}^{l}}=\frac{1}{x_{1}\left(1+N_{22}^{l}\right)}$$

which still yields $N_{22}^{l}+1$ yet with an additional factor, *x*_1_ (see Supporting Information: C). We emphasize that $N_{22}^{l}+1$ has a clear physical meaning as the aggregation number (see eq 21 of ref 64, because it is the sum of $N_{22}^{l}$ (the excess number of co-molecules around a probe co-molecule) and +1 (the number of the probe co-molecule itself)^55^ (note that the molality scale offers a more direct route to the aggregation number, hence will be used throughout this paper).

### Gradient and Curvature of the Surface Tension Isotherm (Tool C)

#### Gradient

Conventionally, surface tension isotherms are plotted against co-molecule concentration. This necessitates a synthesis of our theories on adsorption (eq 7) and bulk self-assembly (eq 13) via

15 $${\left(\frac{\partial \gamma_{s}}{\partial \ln m_{2}}\right)}_{T}={\left(\frac{\partial \gamma_{s}}{\partial \ln a_{2}}\right)}_{T}{\left(\frac{\partial \ln a_{2}}{\partial \ln m_{2}}\right)}_{T}$$

which yields

16 $$-\frac{1}{RT}{\left(\frac{\partial \gamma_{s}}{\partial \ln m_{2}}\right)}_{T}=\frac{\Gamma_{s2}}{1+N_{22}^{l}}$$

Thus, according to eq 16, the gradient of a surface tension isotherm is the competition between

(a) the adsorption of co-molecules (widely observed for surfactants or co-solvents) at the interface, which makes Γ_s2_ positive and drives down the surface tension
(b) the bulk self-assembly of co-molecules (e.g., CMC for surfactants and bulk self-association of alcohols), which makes $N_{22}^{l}$ positive and attenuates the decrease in surface tension due to a

This interpretation (Figure 3) is universal, without involving any model assumptions, thanks to the exact derivations of eq 16.

**Figure 3:**
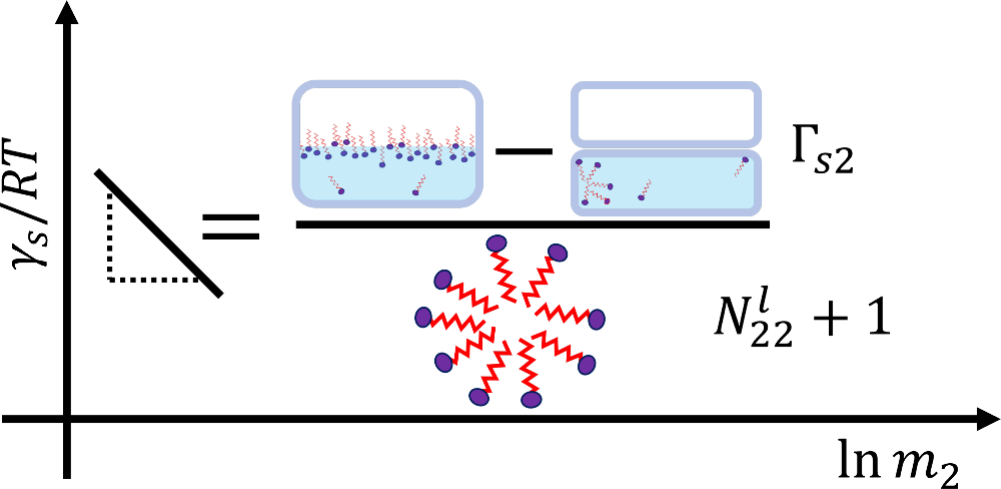
Schematic representation of eq 16. The ln *m*_2_ gradient of the surface tension is determined by a competition between surface excess (Γ_s2_) and bulk self-assembly $N_{22}^{l}+1$ of surfactants.

#### Curvature

The curvature (second-order derivative) of a surface tension isotherm can be expressed as (see Supporting Information: D),

17 $$-\frac{1}{RT}{\left(\frac{\partial^{2}\gamma_{s}}{\partial {\left[\ln m_{2}\right]}^{2}}\right)}_{T}=\frac{1}{{\left(1+N_{22}^{l}\right)}^{2}}\left[\frac{\Delta_{s}\left\langle \delta n_{2}\delta n_{2}\right\rangle }{\sigma }-\Delta_{2}\left\langle \delta n_{2}^{1}\delta n_{2}^{1}\right\rangle \frac{\Gamma_{s2}}{1+N_{22}^{l}}\right]$$

where Δ_2_⟨δ*n*_2_^1^δ*n*_2_^1^⟩, excess fluctuation around the probe co-molecule, is defined as

18 $$\Delta_{2}\left\langle \delta n_{2}^{1}\delta n_{2}^{1}\right\rangle \equiv {\left\langle \delta n_{2}^{1}\delta n_{2}^{1}\right\rangle }_{2}-\left\langle \delta n_{2}^{1}\delta n_{2}^{1}\right\rangle$$

which measures the increase of co-molecule fluctuation around a probe co-molecule ${\left\langle \delta n_{2}^{1}\delta n_{2}^{1}\right\rangle }_{2}$ compared to the bulk $\left\langle \delta n_{2}^{1}\delta n_{2}^{1}\right\rangle$ . Thus, according to eq 17, the key contributors to the curvature are

(a) a positive surface excess fluctuation, Δ_s_⟨δ*n*_2_δ*n*_2_⟩ of eqs 8 and 9, which drives down the second derivative under Γ_s2_ > 0 and drives it up under Γ_s2_ < 0
(b) a positive excess fluctuation around a probe co-solvent, Δ_2_⟨δ*n*_2_^1^δ*n*_2_^1^⟩, which drives up the second derivative
(c) co-solvent self-association in the bulk, $N_{22}^{l}$ , attenuates a and b

We emphasize that eq 17 is an exact relationship, which guarantees the accuracy of its interpretation presented above. Our theory can be extended straightforwardly to electrolyte co-molecules^65,66^ (see Supporting Information: E). Thus, we established a universal interpretation tool for the gradient and curvature of a surface tension isotherm based on exact relationships without employing any models.

### Equations for Surface Tension Isotherms (Tool D)

#### Deriving a Surface Tension Isotherm from an Adsorption Isotherm

Our goal is to derive equations for surface tension isotherms, i.e., how the surface tension changes with co-molecule concentration. This can be achieved straightaway by integrating the generalized Gibbs isotherm (eq 7), which yields

19 $$-\frac{\gamma_{s}-\gamma_{s}^{o}}{RT}=\int_{0}^{a_{2}}\frac{\Gamma_{s2}}{a_{2}^{\prime }}da_{2}^{\prime }$$

where $a_{2}^{\prime }$ is the dummy integration variable for the activity. Consequently, a surface tension isotherm equation can be obtained by integrating a sorption isotherm (i.e., Γ_s2_ on the right-hand side of eq 19). When a clear physical interpretation is attributed to the parameters of Γ_s2_ (see below), the surface tension equation, obtained through eq 19, will attain the same level of interpretive clarity.

Recently, we have demonstrated that a wide class of solid/gas and solid/liquid isotherms can be modeled by two basic isotherms (ABC and cooperative), with two methods for combination (isotherm additivity and multiplicativity), that can all be derived from the fluctuation equation (eqs 8 and 9).^9,61^ In the following, we will focus on the ABC isotherm as a natural generalization of the Szyszkowski–Langmuir model.

#### ABC Isotherm

Here, we generalize (see Supporting Information: F) our recent work on solid/gas and solid/liquid sorption to air/liquid interface.^8,9,53,60,62^ Our starting point is the combination of eqs 8 and 9, which yields

20 $${\left(\frac{\partial }{\partial a_{2}}\frac{a_{2}}{\Gamma_{s2}}\right)}_{T}=-\sigma \frac{\left\langle n_{2}^{*}\right\rangle N_{22}^{*}-\left\langle n_{2}^{l}\right\rangle N_{22}^{l}-\left\langle n_{2}^{a}\right\rangle N_{22}^{a}}{{\left(\left\langle n_{2}^{*}\right\rangle -\left\langle n_{2}^{l}\right\rangle -\left\langle n_{2}^{a}\right\rangle \right)}^{2}}$$

Thus, the only postulates for the ABC isotherm are (i) the (generalized) Gibbs isotherm (for the surface excess Γ_s2_), (ii) the finite ranged nature of the interfacial effect, and (iii) a series expansion of *a*_2_/Γ_s2_. Integrating eq 20 alongside a series expansion^8,63^

21 $$\sigma \frac{\left\langle n_{2}^{*}\right\rangle N_{22}^{*}-\left\langle n_{2}^{l}\right\rangle N_{22}^{l}-\left\langle n_{2}^{a}\right\rangle N_{22}^{a}}{{\left(\left\langle n_{2}^{*}\right\rangle -\left\langle n_{2}^{l}\right\rangle -\left\langle n_{2}^{a}\right\rangle \right)}^{2}}=B_{0}+C_{0}a_{2}$$

yields the ABC isotherm^53,63^ for air/liquid interface

22 $$\Gamma_{s2}=\frac{a_{2}}{A_{0}-B_{0}a_{2}-\frac{C_{0}}{2}a_{2}^{2}}$$

with its parameters defined via

23 $$\begin{array}{l} \frac{1}{A_{0}}={\left(\frac{\Gamma_{s2}}{a_{2}}\right)}_{a_{2}\to 0},\\ B_{0}=\sigma {\left(\frac{\left\langle n_{2}^{*}\right\rangle N_{22}^{*}-\left\langle n_{2}^{l}\right\rangle N_{22}^{l}-\left\langle n_{2}^{a}\right\rangle N_{22}^{a}}{{\left(\left\langle n_{2}^{*}\right\rangle -\left\langle n_{2}^{l}\right\rangle -\left\langle n_{2}^{a}\right\rangle \right)}^{2}}\right)}_{a_{2}\to 0},\\ C_{0}=\sigma {\left(\frac{\partial }{\partial a_{2}}\frac{\left\langle n_{2}^{*}\right\rangle N_{22}^{*}-\left\langle n_{2}^{l}\right\rangle N_{22}^{l}-\left\langle n_{2}^{a}\right\rangle N_{22}^{a}}{{\left(\left\langle n_{2}^{*}\right\rangle -\left\langle n_{2}^{l}\right\rangle -\left\langle n_{2}^{a}\right\rangle \right)}^{2}}\right)}_{a_{2}\to 0} \end{array}$$

In the Results and Discussion, we will demonstrate that $A_{0}^{-1}$ , *B*_0_, and *C*_0_ can be interpreted as sorbate-interface, disorbate, and trisorbate interactions, respectively.^60^ Note that these parameters are defined in terms of number correlations and distributions that are accessible, in principle, to molecular dynamics simulations.^8^ Substituting the ABC adsorption isotherm (eq 22) into eq 19 and carrying out integration^67^ leads to the ABC isotherm for surface tension

24 $$\frac{\gamma_{s}-\gamma_{s}^{o}}{RT}=\frac{2}{\sqrt{-2A_{0}C_{0}-B_{0}^{2}}}\left[\arctan\left(\frac{C_{0}a_{2}+B_{0}}{\sqrt{-2A_{0}C_{0}-B_{0}^{2}}}\right)-\arctan\left(\frac{B_{0}}{\sqrt{-2A_{0}C_{0}-B_{0}^{2}}}\right)\right]$$

which is valid under $B_{0}^{2}<-2A_{0}C_{0}$ . Dependent on the parameter ranges, three other equations emerge for the surface tension isotherm (Table 1) via integration^67^ (eqs 19 with [Disp-formula eq22]). In the Results and Discussion, the ABC isotherm (eq 24) will be applied to fit the ethanol–water and *n*-propanol–water surface tension isotherms, with the mechanistic insights drawn from its parameters.

**Table 1:** Surface Tension Isotherms Derived from the ABC Sorption Isotherm

parameter range	isotherm equation
*C*_0_ = 0	$\frac{\gamma_{s}-\gamma_{s}^{o}}{RT}=\frac{1}{B_{0}}\ln\left(1-\frac{B_{0}}{A_{0}}a_{2}\right)$
*C*_0_ ≠ 0	
$B_{0}^{2}<-2A_{0}C_{0}$	$\frac{\gamma_{s}-\gamma_{s}^{o}}{RT}=\frac{2}{\sqrt{-2A_{0}C_{0}-B_{0}^{2}}}\left(\arctan\frac{C_{0}a_{2}+B_{0}}{\sqrt{-2A_{0}C_{0}-B_{0}^{2}}}-\arctan\frac{B_{0}}{\sqrt{-2A_{0}C_{0}-B_{0}^{2}}}\right)$
$B_{0}^{2}>-2A_{0}C_{0}$	$\frac{\gamma_{s}-\gamma_{s}^{o}}{RT}=-\frac{2}{\sqrt{2A_{0}C_{0}+B_{0}^{2}}}\left(arctanh\frac{C_{0}a_{2}+B_{0}}{\sqrt{2A_{0}C_{0}+B_{0}^{2}}}-arctanh\frac{B_{0}}{\sqrt{2A_{0}C_{0}+B_{0}^{2}}}\right)$ ^a^
$B_{0}^{2}=-2A_{0}C_{0}$	$\frac{\gamma_{s}-\gamma_{s}^{o}}{RT}=\frac{2}{B_{0}}-\frac{2}{B_{0}+C_{0}a_{2}}$

a Expression for $-\sqrt{2A_{0}C_{0}+B_{0}^{2}}$ < $C_{0}a_{2}+B_{0}$ < $\sqrt{2A_{0}C_{0}+B_{0}^{2}}.$ See Supporting Information: F for details.

#### AB Isotherm Generalizes the Szyszkowski–Langmuir Model

Integrating the ABC isotherm leads to another important surface tension isotherm. Under *C*_0_ = 0, integrating^67^ eq 19 with eq 22 yields

25 $$\frac{\gamma_{s}-\gamma_{s}^{o}}{RT}=\frac{1}{B_{0}}\ln\left(1-\frac{B_{0}}{A_{0}}a_{2}\right)$$

which will be referred to as the AB isotherm for surface tension. Conversion from the activity to the concentration scale can be achieved by employing an activity model. The simplest approximation is to retain up to the first order of *x*_2_ for *a*_2_ (expressed in the dilute-ideal reference state),^66^ which leads to the *x*_2_ representation of the AB isotherm

26 $$\frac{\gamma_{s}-\gamma_{s}^{o}}{RT}=\frac{1}{B_{0}}\ln\left(1-\frac{B_{0}}{A_{0}}x_{2}\right)$$

This AB isotherm in the mole-fraction scale (eq 26) is the generalization of the Szyszkowski–Langmuir model^48,49^

27 $$\frac{\gamma_{s}-\gamma_{s}^{o}}{RT}=-n_{m}\ln\left(1+K_{L}x_{2}\right)$$

where *n*_m_ and *K*_L_ are the Langmuir saturation capacity and Langmuir constant, respectively.^8,51^ We emphasize that *n*_m_ and *K*_L_ are assumed to be positive in the Szyzskowski–Langmuir model. A comparison between the Szyszkowski–Langmuir model (eq 27) and the AB isotherm (eq 26) identifies the correspondence between the two, via

28 $$A_{0}=\frac{1}{n_{m}K_{L}},,B_{0}=-\frac{1}{n_{m}}$$

The Szyszkowski–Langmuir model is restricted to site-specific monolayer adsorption on a uniform surface, which does not account for interfacial thickness.^52^ The AB isotherm, in contrast, is free from these restrictions.^8^ Thus, the Szyszkowski–Langmuir model is a special and restricted case of the AB isotherm.

#### Validations for the ABC Isotherm

We have established above that the ABC isotherm is a generalization of the Szyszkowski–Langmuir model via the fluctuation theory. This generalization provides evidence supporting the ABC isotherms. First, the Szyszkowski–Langmuir model can fit the surface tension isotherms for dilute co-molecules (e.g., surfactants^1,3^ and co-solvents^68^). Second, the STAND model, which was successful in modeling surface tension isotherms of surfactants, can be shown as a combination of the Szyszkowski–Langmuir model with a simple model for surfactant aggregation (see Supporting Information: G). Such successful fittings, via eq 28, can be converted to the AB isotherm. Third, the appearance of the arctangent in the ABC isotherm (eq 24) agrees with a recent surface tension model for ethanol–water mixture,^69^ derived based on a simple distribution function assumed for adsorbed molecules.^69,70^ The evidence above justifies the applicability of the ABC isotherm to co-solvents and surfactants alike (see the Results and Discussion for the application to experimental data).

#### Origin of the Saturation Capacity

For the Szyszkowski–Langmuir model (eq 27), the saturation capacity, *n*_m_, is a parameter without any further information on its origin. In contrast, the microscopic origin of the saturation capacity can be revealed by the AB isotherm. For *B*_0_ < 0, the AB sorption isotherm (eq 22 with *C*_0_ = 0) saturates to

29 $$\Gamma_{s2}^{\mathrm{sat}}=-\frac{1}{B_{0}}$$

As is clear from its units, the inverse of $\Gamma_{s2}^{\mathrm{sat}}$ (i.e., $1/\Gamma_{s2}^{\mathrm{sat}}$ ) has been referred to as the “area per molecule”, which is related to *B*_0_ via

30 $$-B_{0}=\frac{1}{\Gamma_{s2}^{\mathrm{sat}}}$$

This paves the way for a more rigorous interpretation of the “area per molecule” (see the Results and Discussion). This can be achieved by combining eq 30 with the definition of *B*_0_ (eq 23). Considering strong adsorption enables us to neglect the bulk reference states in eq 23 via

31 ⟨n2*⟩−⟨n2l⟩−⟨n2a⟩≃⟨n2*⟩⟨n2*⟩N22*−⟨n2l⟩N22l−⟨n2a⟩N22a≃⟨n2*⟩N22*

which simplifies eqs 29 and 30 to

32 $$\frac{1}{\Gamma_{s2}^{\mathrm{sat}}}=-B_{0}=\sigma {\left(\frac{-N_{22}^{*}}{\left\langle n_{2}^{*}\right\rangle }\right)}_{a_{2}\to 0}$$

Thus, saturation takes place when $N_{22}^{*}<0$ , i.e., the net-exclusion of co-molecules around a probe co-molecule. The AB isotherm (putting *C*_0_ onward as zero) means that $N_{22}^{*}/\left\langle n_{2}^{*}\right\rangle$ remains constant independent of co-molecule concentrations. In the Results and Discussion, we will clarify the novel physical insights that emerge from eq 32.

## Results and Discussion

In the following 4 subsections, we will accomplish the 4 objectives listed at the end of the Aims and Objectives of the Introduction.

### Reconceptualizing Aggregation Numbers via the Fluctuation Theory (Objective I)

#### Scope

We will demonstrate that the statistical thermodynamic fluctuation theory

(I-1) gives a model-free definition of the aggregation number
(I-2) clarifies how (1) manifests both in the surface tension isotherm and activity measurements
(I-3) shows the importance of combining activity and surface tension for probing surfactant interactions above CMC

#### Redefining the Aggregation Number (Scope I-1)

Our proposal is to replace the stoichiometric surfactant aggregation models with the statistical thermodynamic fluctuation theory that captures adsorption at the interface and self-association in the bulk solution in the common language of number correlations and fluctuations (see the Theory). This necessitates the reconceptualization of the surfactant aggregation number, *N*_agg_, via

33 $$N_{\mathrm{agg}}=N_{22}^{l}+1=\frac{\left\langle \delta n_{2}^{l}\delta n_{2}^{l}\right\rangle }{\left\langle n_{2}^{l}\right\rangle }$$

­(see Supporting Information: E for the generalization of eq 33 to electrolyte co-molecules). Note that both expressions involve the surfactant excess number around a probe solute (*N*_22_) plus the number of probe surfactant itself (i.e., +1). In the following, we will show that the conventional definition of the aggregation number is a special case of *N*_agg_.

#### Aggregation Number Manifests in Surface Tension Isotherms (Scope I-2)

The abrupt change in gradient of a surface tension isotherm at CMC (see Figure 1) has been used to estimate the surfactant aggregation number.^6,7,71,72^ Here, we demonstrate how an averaged *N*_agg_ (over a concentration range above CMC) manifests both in the surface tension isotherm and the activity measurements. This can be achieved by translating Rusanov’s^6,7^ method of determining *N*_agg_ to our theory, which will establish a more direct route to *N*_agg_, for nonelectrolytes below, with a generalization to electrolytes in Supporting Information: E. As a first step, Rusanov^6,7^ identified the two linear regions in the surface tension isotherm: (i) the linear region (denoted by “lin”) below CMC and (ii) the plateau region (denoted as “plt”) above CMC (Figure 4a). These two gradients are eq 16 averaged over the two concentration ranges of the gradients (denoted by the subscripts):

34 $$\begin{array}{l} -\frac{1}{RT}{\left(\frac{\partial \gamma_{s}}{\partial \ln m_{2}}\right)}_{T;\mathrm{lin}}={\left(\frac{\Gamma_{s2}}{1+N_{22}^{l}}\right)}_{\mathrm{lin}}\\ -\frac{1}{RT}{\left(\frac{\partial \gamma_{s}}{\partial \ln m_{2}}\right)}_{T;\mathrm{plt}}={\left(\frac{\Gamma_{s2}}{1+N_{22}^{l}}\right)}_{\mathrm{plt}} \end{array}$$

**Figure 4:**
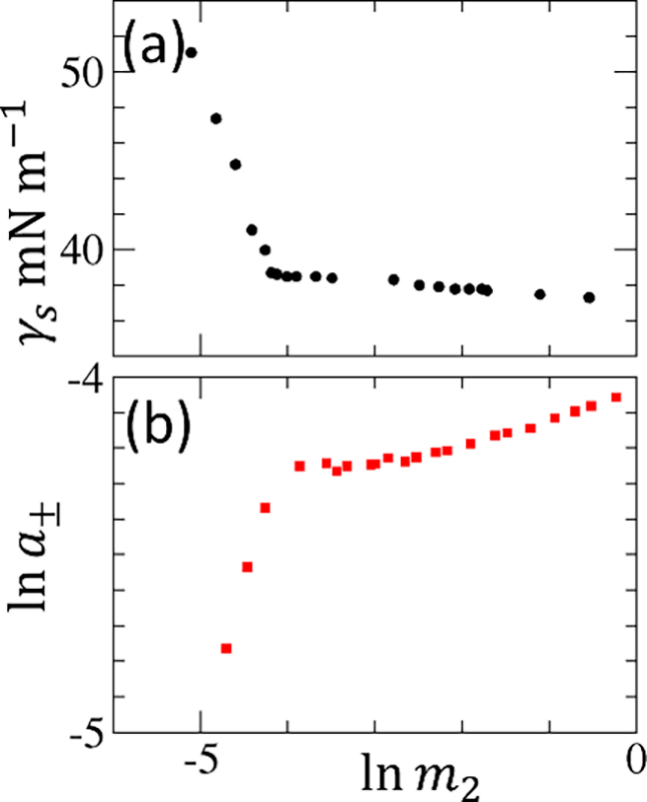
(a) Surface tension isotherm of aqueous dodecyltrimethylammonium bromide (DTAB) solutions at 293 K plotted against ln *m*_2_ of DTAB, by converting the molarity scale in the experimental data published by Prokhorov and Rusanov^73^ to molality using the density data published by Yamanaka and Kaneshina.^74^ (b) Logarithmic DTAB activity (mean ionic activity, see Supporting Information: E for generalization to electrolytes), ln *a*_±_, plotted against ln *m*_2_ using the published activity data at 298 K by De Lisi et al.^75^

Hence, the gradient ratio can be expressed as

35 $$\frac{{\left(\frac{\partial \gamma_{s}}{\partial \ln m_{2}}\right)}_{T;\mathrm{lin}}}{{\left(\frac{\partial \gamma_{s}}{\partial \ln m_{2}}\right)}_{T;\mathrm{plt}}}=\frac{{\left(\frac{\Gamma_{s2}}{1+N_{22}^{l}}\right)}_{\mathrm{lin}}}{{\left(\frac{\Gamma_{s2}}{1+N_{22}^{l}}\right)}_{\mathrm{plt}}}$$

Rusanov^6,7^ assumed that

(i) surfactant aggregation is negligible in the linear region, such that ${\left(1+N_{22}^{l}\right)}_{\mathrm{lin}}≃1$

(ii) the interface is saturated in both regions, such that ${\left(\Gamma_{s2}\right)}_{\mathrm{lin}}≃{\left(\Gamma_{s2}\right)}_{\mathrm{plt}}$ that are both constants
(iii) the isotherm gradient can be calculated from the two averages, i.e., ${\left(\Gamma_{s2}\right)}_{\mathrm{plt}}$ and ${\left(1+N_{22}^{l}\right)}_{\mathrm{plt}}$

where assumption iii was implicit in Rusanov, which nevertheless leads to the following important relationship:

36 $${\left(\frac{\Gamma_{s2}}{1+N_{22}^{l}}\right)}_{\mathrm{plt}}=\frac{{\left(\Gamma_{s2}\right)}_{\mathrm{plt}}}{{\left(1+N_{22}^{l}\right)}_{\mathrm{plt}}}$$

Consequently, under these assumptions, eq 35 simplifies to

37 $$N_{\mathrm{agg}}={\left(1+N_{22}^{l}\right)}_{\mathrm{plt}}≃\frac{{\left(\frac{\partial \gamma_{s}}{\partial \ln m_{2}}\right)}_{T;\mathrm{lin}}}{{\left(\frac{\partial \gamma_{s}}{\partial \ln m_{2}}\right)}_{T;\mathrm{plt}}}$$

We emphasize that the rightmost side of eq 37 is identical to Rusanov’s procedure for estimating the aggregation number.^6,7^ This has two important ramifications. First, the traditional aggregation number, *N*_agg_, has acquired a statistical thermodynamic interpretation in terms of number correlations and excess numbers [see Redefining the Aggregation Number (Scope I-1)]. Second, the alternative route to determine *N*_agg_ results from this generalization. Note that Rusanov’s method (eq 37) involves the evaluation of two isotherm gradients, as well as introducing three assumptions (i–iii) on the unmeasurable quantities (Figure 4a). In contrast, the same *N*_agg_ can be determined solely from the activity measurement (or from the osmotic coefficient itself, see Supporting Information: H) simply via

38 $$N_{\mathrm{agg}}={\left(1+N_{22}^{l}\right)}_{\mathrm{plt}}≃\frac{1}{{\left(\frac{\partial \ln a_{2}}{\partial \ln m_{2}}\right)}_{T,P;\mathrm{plt}}}$$

which involves only one gradient evaluation (Figure 4b). We emphasize that the activity route to *N*_agg_ is free of the three assumptions (i–iii). Thus, evaluating the aggregation number via activity (eq 38) is a more direct route than via surface tension (eq 37). Moreover, it paves the way to examine whether the assumptions (i–iii) made by the surface tension route are valid.

#### Importance of Activity for Probing Surfactant Adsorption and Self-Assembly above CMC (Scope I-3)

Here, we demonstrate the importance of activity when elucidating surfactant self-assembly and surfactant-surface interaction above CMC. To this end, we take dodecyltrimethylammonium bromide (DTAB) as an example, due to the availability of surface tension isotherm data^73^ (Figure 4a) and activity (Figure 4b) data below CMC, which is rare.^75^ For this system, mean ionic activity must be employed (see Supporting Information: E and H). Both surface tension and activity exhibit a clear break at CMC when plotted against ln *m*_2_. Above CMC, however, we arrive at an apparent contradiction:

(a) the gradient of the surface tension isotherm, plotted against ln *m*_2_, remains virtually constant (Figure 4a), implying (via assumption ii and eq 36) a constant *N*_agg_ above CMC
(b) the gradient of ln *a*_2_ increases above CMC (Figure 4b), implying (via eq 13) that *N*_agg_ decrease above CMC

To resolve this contradiction, assumption ii (i.e., the constancy of Γ_s2_ above CMC) must be re-examined. To achieve this, employing ln *a*_2_ as the variable gives us a clearer understanding of both Γ_s2_ (via eq 4) and *N*_agg_ (via eq 38):

(a) Γ_s2_, which is the gradient of Figure 5a, is still positive above CMC (due to the compression in the range of ln *a*_2_ compared to ln *m*_2_), yet with a reduced magnitude
(b) *N*_agg_, which is the gradient of Figure 5b, is large above CMC (between b and c of Figure 5b) yet decreases at higher surfactant concentrations (above c of Figure 5b)

**Figure 5:**
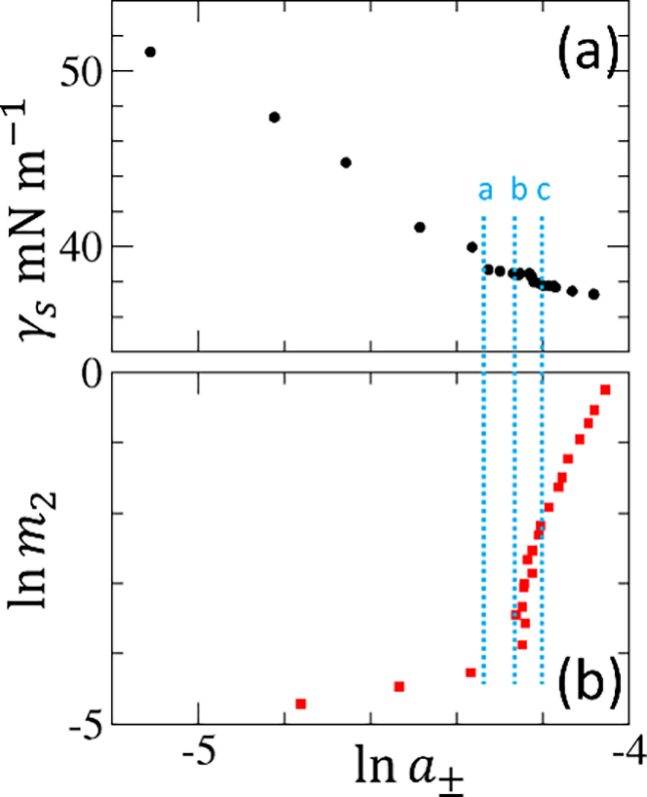
(a) Surface tension isotherm of aqueous dodecyltrimethylammonium bromide (DTAB) solutions at 293 K plotted against ln *a*_2_ of DTAB, by converting the molality scale in Figure 4a to *a*_2_ using the activity data in Figure 4b using cubic spline interpolation. (b) Logarithmic DTAB molality, ln *m*_2_, plotted against the mean ionic activity of DTAB ln *a*_±_ based on Figure 4b.

Consequently, both Γ_s2_ and *N*_agg_ decrease above CMC. This observation can be rationalized by the definitions of Γ_s2_ and *N*_agg_. According to the definition of surface excess (eq 4), while the “saturation” at the interface may keep $\left\langle n_{2}^{*}\right\rangle$ unchanged above CMC, $\left\langle n_{2}^{l}\right\rangle$ in the bulk solution keeps increasing, thereby leading to an overall decrease of Γ_s2_. Likewise, $N_{22}^{l}$ in the aggregation number is defined by eq 5, which involves a difference between the surfactant number around a probe surfactant $\left({\left\langle n_{2}^{l}\right\rangle }_{2}\right)$ and the bulk $\left(\left\langle n_{2}^{l}\right\rangle \right)$ . The surfactant concentration automatically increases the bulk $\left\langle n_{2}^{l}\right\rangle$ , making the solution more homogeneous, making the presence of the probe surfactant less special (note that the activity will be crucial for filling the remaining gap between the fluctuation theory and the previous approaches that employ stoichiometric aggregation models;^41^ see Supporting Information: G). Thus, we have demonstrated that a combination of the surface tension isotherm and surfactant activity is crucial for probing surfactant interactions above the CMC.

### Unifying Surfactants and Non-surfactants (Objective II)

#### Scope

Here, we demonstrate that our theory is applicable to surfactants and non-surfactants alike, taking aqueous alcohol solutions, known to exhibit complex self-association behavior, as examples. We will

(II-1) resolve the apparent CMC paradox^59^ for aqueous alcohol mixtures
(II-2) attribute the plateau in surface tension isotherms to bulk aggregation (eq 16)
(II-3) demonstrate the superiority of the activity data to surface tension isotherms in detecting aggregation in the bulk

#### Resolving the Apparent CMC Paradox in Propanol–Water Mixtures (Scope II-1)

Here, we present a resolution of the apparent CMC mystery in *n*-propanol–water mixture.^59^ This can be achieved by identifying the precise reasons behind the CMC-like behavior in the ln *x*_2_ plot (Figure 2a), in contrast to the lack thereof in ln *a*_2_ plot (Figure 2b).^59^ The two gradients are linked via ${\left(\frac{\partial \ln a_{2}}{\partial \ln x_{2}}\right)}_{T,P}$ hence the CMC-like behavior should be attributed to the plateau in the plot of ln *a*_2_ against ln *x*_2_ (Figure 6), whose gradient, via eq 14, is $\frac{1}{x_{1}\left(N_{22}^{l}+1\right)}$ .

**Figure 6:**
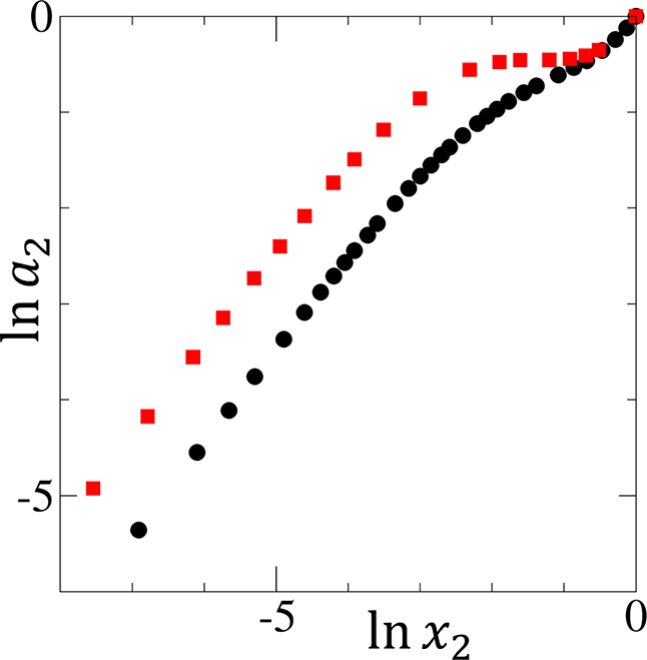
Logarithmic activity (ln *a*_2_) against the logarithmic mole fraction (ln *x*_2_) of ethanol (black circles) and *n*-propanol (red squares) in their aqueous solutions, using the published activity data at 25 °C by Strey et al.^2^ The origin of CMC-like behavior (see Figure 2) is caused by the near-zero gradient of ${\left(\frac{\partial \ln a_{2}}{\partial \ln x_{2}}\right)}_{T,P}$ at ln *x*_2_ > −2.5 for the *n*-propanol–water mixture (red squares), while the lack of such behavior for ethanol–water mixture (black circles) is due to ${\left(\frac{\partial \ln a_{2}}{\partial \ln x_{2}}\right)}_{T,P}>0$ for the entire concentration range.

#### Attributing the Plateau in Surface Tension Isotherms to Bulk Aggregation (Scope II-2)

In the molality scale, the plateau in the surface tension isotherm (Figure 7a) is attributed to a large $N_{22}^{l}+1$ (Figure 7b, whose gradient is $\frac{1}{N_{22}^{l}+1}$ , via eq 13). However, the plateau takes place at high *x*_2_, which means the bulk liquid subsystem contains more alcohol molecules than water. Consequently, unlike the case of surfactants, $N_{22}^{l}+1$ cannot be interpreted simply as *N*_agg_, i.e., the aggregation number of alcohols. In this case, it would be more convenient to treat the solvent and co-molecule in a symmetrical manner, in the {*T*, *v* ^l^, μ_1_, μ_2_} ensemble. An ensemble transformation^64,76^ $\left\{T,P,n_{1}^{l},\mu_{2}\right\}\to \left\{T,v^{l},\mu_{1},\mu_{2}\right\}$ (Supporting Information: I) allows $N_{22}^{l}+1$ to be interpreted as the measure of solution segregation (i.e., alcohol–alcohol and water–water interactions stronger than alcohol–water) using the Kirkwood–Buff integrals. Thus, a large $N_{22}^{l}+1$ is the key to the plateau in the surface tension isotherms for surfactants and non-surfactants.

**Figure 7:**
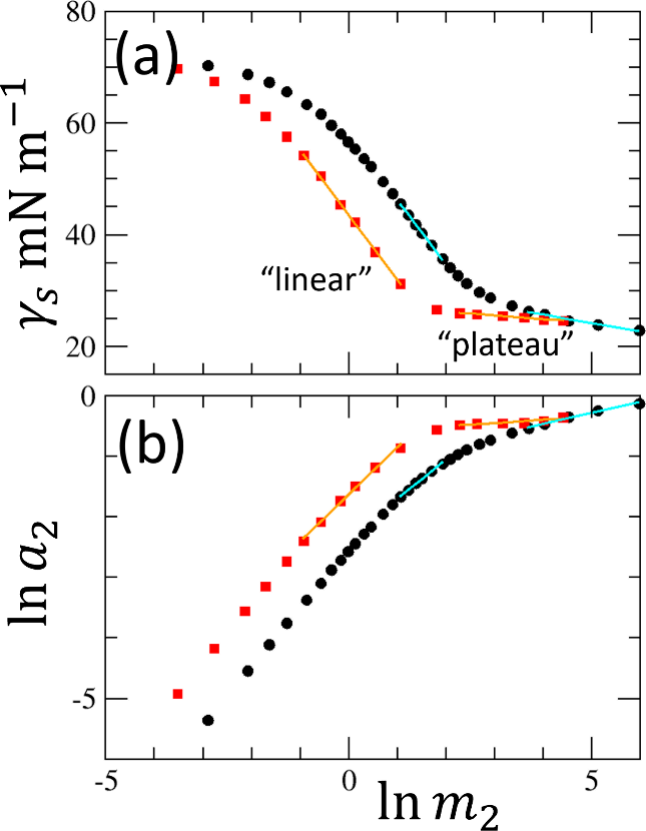
(a) Surface tension isotherm of water–ethanol (black circles and cyan lines) and water–*n*-propanol mixtures (red squares and orange lines) replotted against the logarithmic molality scale of alcohols (ln *m*_2_) in conformity to our theory (eq 16). Analogous to Figure 4 for DTAB, the “linear” and “plateau” regions have been identified via visual inspection, indicated by the orange and cyan solid lines. (b) Plot of ln *a*_2_ against ln *m*_2_ for the same mixtures, whose gradient yields $1/\left(N_{22}^{l}+1\right)$ via eq 13, with the linear regressions in orange and cyan lines for the “linear” and “plateau” regions defined in panel a. For ethanol and *n*-propanol, $N_{22}^{l}+1$ in the plateau region is estimated to be ∼5.5 and 20, respectively.

#### Superiority of Activity over Surface Tension Isotherm (Scope II-3)

Unlike the observation of *n*-propanol self-association via the surface tension isotherm (Figure 7a) and activity (Figure 7b), it is difficult to conclude solely from the surface tension isotherm, replotted choosing alcohol as solvent, water as co-molecule (Figure 8a), whether its near-constancy is due to an ambivalent surface excess of water (Γ_s2_ ≃ 0; neither surface accumulation nor exclusion) or strong segregation of solvent mixtures (i.e., a large $N_{22}^{l}$ ). On the contrary, the activity plot (Figure 8b) clearly indicates a plateau, demonstrating the segregation of *n*-propanol and water, but not water and ethanol. This underscores our lesson from surfactants: the activity plot is more versatile for identifying segregation in bulk solutions.

**Figure 8:**
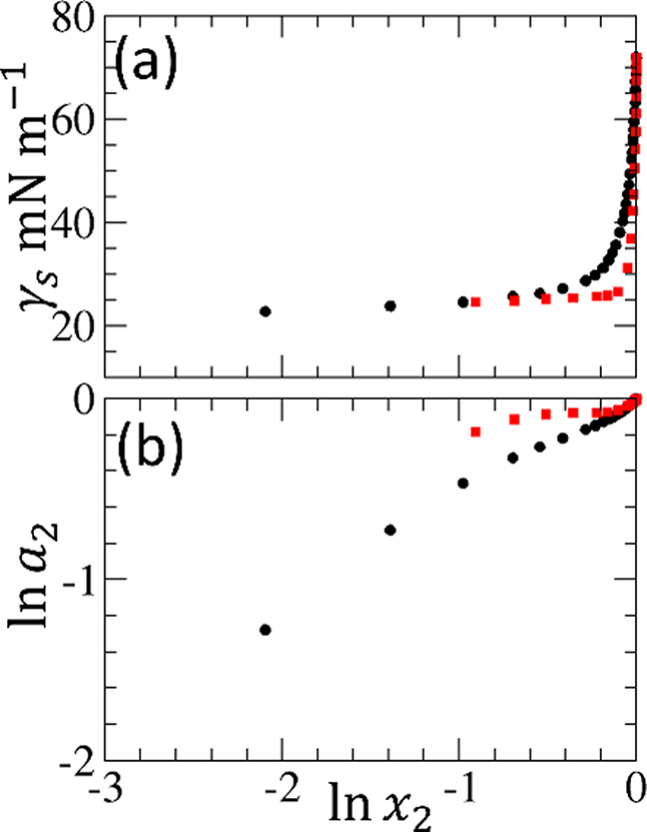
(a) Surface tension isotherms of ethanol–water (black circles) and *n*-propanol–water (red squares) mixtures, denoted with 1 = alcohol and 2 = water, replotted against ln *x*_2_ (where *x*_2_ is the mole fraction of water). (b) Logarithmic activity (ln *a*_2_) against the ln *x*_2_ of water dissolved in ethanol (black circles) and *n*-propanol (red squares) solvents, using the published activity data at 25 °C by Strey et al.^59^ Because of the very gradual rise in the isotherm, it is difficult to tell from the isotherm (a) whether it is due to weak adsorption or by alcohol self-aggregation as clearly as one could conclude from the activity plot (b).

### Establishing Equations for Surface Tension Isotherm with Their Parameters Representing the Underlying Interactions (Objective III)

#### Scope

Unlike the previous models, our goal is to employ minimum model assumptions to derive equations for surface tension isotherms so that

(III-1) the Szyszkowski–Langmuir model (restricted to adsorption without layer thickenss) be replaced with the AB isotherm
(III-2) the ABC isotherm can capture attractive and repulsive surface–co-molecule and co-molecule–co-molecule interactions
(III-3) the adsorption capacity at saturation can be interpreted by the projected area of co-molecules, replacing the previous “area-per-molecule” that did not consider layer thickness^52^

#### AB Isotherm Replaces the Szyszkowski–Langmuir Model (Scope III-1)

Here, we show that the ABC isotherm (eq 24), or even the AB isotherm (eq 25) as its special case, enjoys much wider applicability than the Szyszkowski–Langmuir model (eq 27).^48,49^ This can be appreciated most intuitively by comparing the Maclaurin expansion of the Szyszkowski–Langmuir model (with *a*_2_ as its variable) with that of the AB isotherm

39 $$\begin{array}{l} \frac{\gamma_{s}-\gamma_{s}^{o}}{RT}=-n_{m}K_{L}a_{2}+\frac{n_{m}K_{L}^{2}}{2}a_{2}^{2}+\cdot \cdot \cdot \end{array}$$

40 $$\begin{array}{l} \frac{\gamma_{s}-\gamma_{s}^{o}}{RT}=-\frac{1}{A_{0}}a_{2}-\frac{B_{0}}{2A_{0}^{2}}a_{2}^{2}+\cdot \cdot \cdot \end{array}$$

­(note that eq 40 can also be derived straightaway from the polynomial isotherm;^62^ see Supporting Information: F). As is clear from its form (eq 27), the Szyszkowski–Langmuir model can only be applied to decreasing surface tension, with a convex functional shape, with co-molecule concentration; this is because *n*_m_ (the “number of binding sites”) and *K*_L_ (“binding constant”) must both be positive in the Szyszkowski–Langmuir model.^8^ In contrast, the AB isotherm is free from such restrictions, applicable equally to increasing and decreasing surface tension, and to convex and concave functional shapes, because of its ability to handle^8^

- both repulsive (*A*_0_ < 0) and attractive (*A*_0_ > 0) surface–co-molecule interaction
- both strengthened (*B*_0_ > 0) and weakened (*B*_0_ < 0) co-molecule–co-molecule interaction

Thus, the AB isotherm has eliminated the idealized assumptions of the Szyszkowski–Langmuir model. The AB isotherm’s ability to handle attractive and repulsive interactions alike is the reason why the AB isotherm enjoys wider applicability than the Szyszkowski–Langmuir model.

#### ABC Isotherm Captures Attractive and Repulsive Surface–Co-molecule and Co-molecule–Co-molecule Interactions (Scope III-2)

The ABC isotherm (eq 24) can capture the surface tension isotherm along the concentration of alcohols (1 = water, 2 = alcohol; Figure 9) and the concentration of water (1 = alcohol, 2 = water; Figure 10). Upon the addition of alcohol to water (Figure 9), a strong alcohol adsorption $\left(\left\langle n_{2}^{*}\right\rangle \gg \left\langle n_{2}^{l}\right\rangle ,\left\langle n_{2}^{a}\right\rangle \right)$ leads to a simplification in the interpretation of the parameters

41 $$\begin{array}{l} \frac{1}{A_{0}}≃\frac{1}{\sigma }{\left(\frac{\left\langle n_{2}^{*}\right\rangle }{a_{2}}\right)}_{a_{2}\to 0},\\ B_{0}≃\sigma {\left(\frac{N_{22}^{*}}{\left\langle n_{2}^{*}\right\rangle }\right)}_{a_{2}\to 0},\\ C_{0}≃\sigma {\left(\frac{\partial }{\partial a_{2}}\frac{N_{22}^{*}}{\left\langle n_{2}^{*}\right\rangle }\right)}_{a_{2}\to 0} \end{array}$$

**Figure 9:**
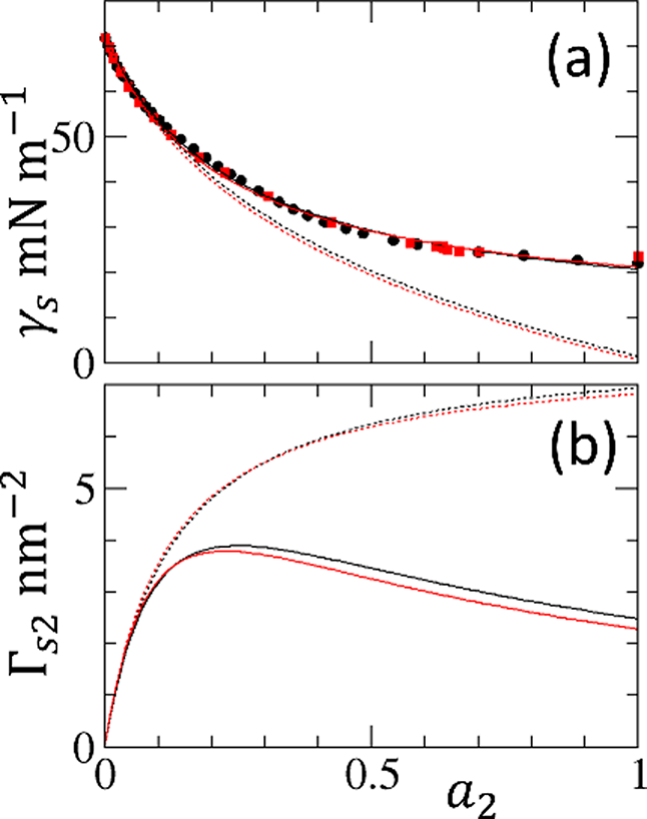
(a) Application of the ABC (solid lines) and AB (dotted lines) isotherms to fit the experimental surface tension data of water–ethanol (black circles) and water–*n*-propanol (red squares) mixtures against the logarithmic alcohol activity, ln *a*_2_ (see Figure 2). (b) Corresponding adsorption isotherms from the ABC (solid lines) and AB isotherms. The fitting parameters $\left(A_{0},B_{0},C_{0}\right)$ in nm^2^ are (0.0161, −0.128, −0.517) for water–ethanol and (0.0151, −0.132, −0.584) for water–*n*-propanol mixtures.

**Figure 10:**
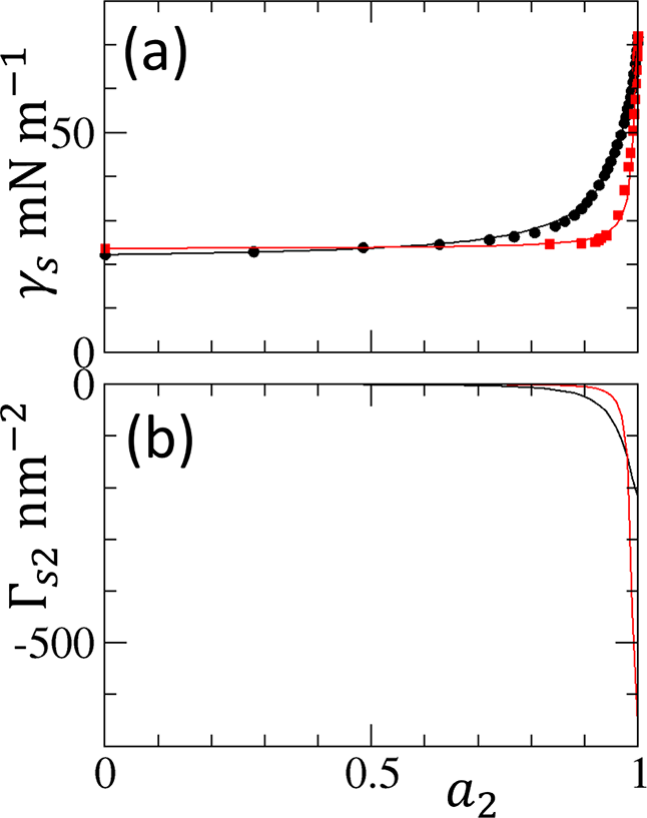
(a) Application of the ABC (solid lines) isotherm to fit the experimental surface tension data of ethanol–water (black circles) and *n*-propanol–water (red squares) mixtures against the logarithmic water activity, ln *a*_2_. (b) Corresponding adsorption isotherms from the ABC (solid lines) isotherm. The fitting parameters $\left(A_{0},B_{0},C_{0}\right)$ in nm^2^ are (2.75, −5.43, 5.37) for ethanol–water and (19.5, −39.1, 39.3) for *n*-propanol–water mixtures.

Consequently, the negative *B*_0_ signifies alcohol–alcohol exclusion at the interface and the negative *C*_0_ indicates the weakening alcohol–alcohol interaction via 3-body repulsion (since these parameters were determined by fitting the ABC isotherm to the experimental surface tension isotherm, the *a*_2_ → 0 in eq 41 should signify extrapolation rather than actual measurements carried out at this limit; see also the note below eq F.4 of the Supporting Information). Upon the addition of water to alcohols (Figure 10), the weak exclusion of water from the interface is indicated by a small, negative $A_{0}^{-1}$ , yet the exclusion becomes prominent at higher water concentrations. Consequently, the interpretation for the negative *B*_0_ and positive *C*_0_ can be provided by eq 21 rather than eq 41. Under a strong exclusion of co-molecules at the interface, $\left\langle n_{2}^{l}\right\rangle \gg \left\langle n_{2}^{*}\right\rangle ,\left\langle n_{2}^{a}\right\rangle$ , eq 21 behaves at high water concentration region, as

42 $$\sigma \frac{N_{22}^{l}}{\left\langle n_{2}^{l}\right\rangle }=-B_{0}-C_{0}a_{2}+\cdot \cdot \cdot$$

Thus, *B*_0_ and *C*_0_ represent a strong (a large, negative *B*_0_) but attenuating (a large, positive *C*_0_) water self-association in the bulk as the water concentration increases. In the case of surface exclusion, the bulk self-association of water molecules drives the shape of the surface tension isotherm. Thus, we have demonstrated that the ABC isotherm can capture the attractive and repulsive surface–co-molecule and co-molecule–co-molecule interactions at the interface and in the bulk.

#### Incorporating Adsorption Layer Thickness (Scope III-3)

Is the Gibbs analysis of surface tension questionable because of the unintuitive values obtained for the saturating capacity, $\Gamma_{s2}^{\mathrm{sat}}$ , or the “area-per-molecule”, $1/\Gamma_{s2}^{\mathrm{sat}}$ , as its inverse? No further information can be obtained from the Szyszkowski–Langmuir model about the nature of $n_{m}=\Gamma_{s2}^{\mathrm{sat}}$ . Such aporia can be overcome by the AB isotherm, as we demonstrated here. Combining the AB isotherm expression for $1/\Gamma_{s2}^{\mathrm{sat}}$ (eq 30) for strongly adsorbing co-molecules with the definition of the excess number (eq 4), we obtain

43 $$\frac{1}{\Gamma_{s2}^{\mathrm{sat}}}=-B_{0}=\sigma {\left(\frac{\left\langle n_{2}^{*}\right\rangle -{\left\langle n_{2}^{*}\right\rangle }_{2}}{\left\langle n_{2}^{*}\right\rangle }\right)}_{a_{2}\to 0}$$

through eqs 5 and 41. Here, we introduce the excluded volume (or covolume), $v_{2}^{*}$ , via

44 $$c_{2}^{*}v_{2}^{*}=\left\langle n_{2}^{*}\right\rangle -{\left\langle n_{2}^{*}\right\rangle }_{2}$$

where $c_{2}^{*}=\left\langle n_{2}^{*}\right\rangle /v^{*}$ is the co-molecule concentration in the interface with v* being the volume of the interface noted in the next paragraph. $v_{2}^{*}$ signifies the net volume around a probe co-molecule which other co-molecules cannot enter. Using $c_{2}^{*}=\left\langle n_{2}^{*}\right\rangle /v^{*}$ , eqs 43 and 44 transform to

45 $$\frac{1}{\Gamma_{s2}^{\mathrm{sat}}}≃{\left(\frac{v_{2}^{*}}{\delta^{*}}\right)}_{a_{2}\to 0}$$

with the interfacial thickness introduced by

46 $$\delta^{*}=\frac{v^{*}}{\sigma }$$

where σ is the area of the interface introduced at the beginning of the Theory.

Thus, our proposal is to replace the “area-per-molecule” ^10,43^ with what we term the “projected area”, $1/\Gamma_{s2}^{\mathrm{sat}}$ . Without taking into account the thickness of the interfacial layer, $\Gamma_{s2}^{\mathrm{sat}}$ is inevitably interpreted as the adsorbed molecule per unit area of a surface (without thickness). It follows that $1/\Gamma_{s2}^{\mathrm{sat}}$ signifies area-per-molecule. Our theory, in contrast, considers interfacial thickness, hence the same quantity, $1/\Gamma_{s2}^{\mathrm{sat}}$ , acquires a new interpretation via eq 45 as the excluded volume divided by the thickness of the interface. We emphasize that the projected area itself is determined solely by the saturation capacity, $\Gamma_{s2}^{\mathrm{sat}}$ , which is experimentally measurable. For its theoretical interpretation via the projected area (i.e., the right-hand side of eq 45), both $v_{2}^{*}$ and δ* depend on the evaluation of v*. This can be carried out (with the help of molecular dynamics simulations^77^) by setting the boundary between the interface and the bulk through the convergence of local co-molecule concentration to its bulk solution limit. Consequently, the volume v* is dependent inevitably on the definition of convergence, yet does not affect $v_{2}^{*}/\delta^{*}$ itself.

In contrast, capturing interfacial thickness was beyond the reach of the two-dimensional nature of the Szyszkowski–Langmuir model.^52^ The two-dimensional picture of $1/\Gamma_{s2}^{\mathrm{sat}}$ has led to the historical paradox that the area per surfactant is too large and insensitive to the molecular structures of the surfactant.^10,43,44^ Resolution to this paradox comes from our new interpretation of $1/\Gamma_{s2}^{\mathrm{sat}}$ as the projected area (eq 45): a larger surfactant has not only a larger excluded volume, but also a larger interfacial thickness. Thus, the key to elucidating the projected area lies in a balance between the excluded volume and the thickness of the interface. We emphasize that quantifying these two determining factors requires careful study through simulation.

### Linking Surfactant Isotherm Features to Interactions (Objective IV)

#### Scope

Now we return to our goal of elucidating the interactions underlying the surface tension isotherm (Figure 1). We have already shown that

- bulk self-assembly is responsible for the plateau
- the aggregation number can change above CMC

In this section, armed with the interpretive tools for the gradient (eq 16) and curvature (eq 17) of the surface tension isotherms, we will clarify the interactions underlying

(IV-1) the “precipitous decline”^10^
(IV-2) the near-linear region of the surface tension isotherm
(IV-3) premicelle formation

#### “Precipitous Decline” of Surface Tension (Scope IV-1)

We present here a resolution to the paradox surrounding the sudden onset of surface tension decline, i.e., “why the surface tension remain(s) relatively constant in region A [in Figure 1] but then begin its precipitous decline only after saturation is ostensively reached at the beginning of region B”.^10^ As the first step, we can safely neglect surfactant self-association in this dilute concentration range, such that $N_{22}^{l}+1≃1$ . Consequently, we can employ the AB isotherm for surface tension of eq 26. Since $\gamma_{s}-\gamma_{s}^{o}$ is small at the onset of its decline, eq 26 can be expanded, leading to

47 $$-\frac{\gamma_{s}-\gamma_{s}^{o}}{RT}≃\frac{x_{2}}{A_{0}}≃\frac{m_{2}}{M_{1}A_{0}}$$

In this concentration range, surface tension is dominated by the surface–surfactant interaction, $A_{0}^{-1}$ . Conforming to the convention of plotting the surface tension isotherms against ln *m*_2_, eq 47 converts to

48 $$-\frac{\gamma_{s}-\gamma_{s}^{o}}{RT}≃\frac{x_{2}}{A_{0}}≃\frac{1}{M_{1}A_{0}}e^{\ln m_{2}}$$

According to eq 47, $\gamma_{s}-\gamma_{s}^{o}$ changes linearly with *m*_2_. In contrast, when we plot $\gamma_{s}-\gamma_{s}^{o}$ against ln *m*_2_, not only is the region A (*m*_2_ ≪ 1) stretched but also with a very small gradient [*m*_2_/(*M*_1_ *A*_0_)] according to eq 48. Thus, the apparent “precipitous decline”^10^ is an artifact of the logarithmic plot, which is exaggerated especially for the low surfactant concentration region.

#### Near-Linear Region as Adsorption Capacity at Saturation (Scope IV-2)

Considering the surfactant self-association that is still negligible (i.e., $N_{22}^{l}+1≃1$ ) in this region, eq 16, becomes

49 $$-\frac{1}{RT}{\left(\frac{\partial \gamma_{s}}{\partial \ln m_{2}}\right)}_{T}≃\Gamma_{s2}$$

This means that Γ_s2_ is the ln *m*_2_ gradient of the surface tension isotherm. Consequently, a near-linear decline of the surface tension isotherm signifies the near-constancy of Γ_s2_,^47^ or its approach to the saturation capacity

50 $$\Gamma_{s2}≃\Gamma_{s2}^{\mathrm{sat}}$$

This rules out^47^ the proposal that “the surface tension decline in region B arises from a continuously increasing occupancy of the interface”^10^ because increasing Γ_2s_, according to eq 16, leads to a continuous steepening of the surface tension isotherm rather than its convergence (see Supporting Information: J for the agreement of our explanation with Rosen and Kunjappu^1^). Thus, we have shown via eqs 49 and 50 that the near-saturation of Γ_s2_ leads to a constant decrease in the surface tension isotherm plotted against the logarithmic concentration.

#### Premicelles (Scope IV-3)

Here, we rationalize the existence of premicelles (see the Introduction) and variability in the aggregation number around CMC.^7^ Above the linear region, surface adsorption is saturated. Hence the gradient of the surface tension (eq 16) simplifies to

51 $$-\frac{1}{RT}{\left(\frac{\partial \gamma_{s}}{\partial \ln m_{2}}\right)}_{T}=\frac{\Gamma_{s2}^{\mathrm{sat}}}{1+N_{22}^{l}}$$

Consequently, a break in the surface tension isotherm gradient comes from the abrupt change in $1+N_{22}^{l}$ . This gradient change is quantified by the curvature (second-order derivative) of the surface tension isotherm, which can be expressed (see Supporting Information: D) as

52 $$\frac{1}{RT}{\left(\frac{\partial^{2}\gamma_{s}}{\partial {\left[\ln m_{2}\right]}^{2}}\right)}_{T}=\Gamma_{s2}^{\mathrm{sat}}\frac{\Delta_{2}\left\langle \delta n_{2}^{l}\delta n_{2}^{l}\right\rangle }{{\left(1+N_{22}^{l}\right)}^{3}}$$

with $\Delta_{2}\left\langle \delta n_{2}^{l}\delta n_{2}^{l}\right\rangle$ expressing the enhancement of surfactant number fluctuation around a surfactant. Before surfactant aggregation, $N_{22}^{l}+1≃1$ , hence eq 52 simplifies to

53 $$\frac{1}{RT}{\left(\frac{\partial^{2}\gamma_{s}}{\partial {\left[\ln m_{2}\right]}^{2}}\right)}_{T}=\Gamma_{s2}^{\mathrm{sat}}\Delta_{2}\left\langle \delta n_{2}^{l}\delta n_{2}^{l}\right\rangle$$

Consequently, around a surfactant, surfactant–surfactant number correlation increases ( $\Delta_{2}\left\langle \delta n_{2}^{l}\delta n_{2}^{l}\right\rangle >0$ ), which attenuates the gradient of the surface tension isotherm. This leads to the cooperativity of micelle formation, via the following equation (see Supporting Information: D):

54 $${\left(\frac{\partial {\left[N_{\mathrm{agg}}\right]}^{2}}{\partial \ln m_{2}}\right)}_{T}=2\Delta_{2}\left\langle \delta n_{2}^{l}\delta n_{2}^{l}\right\rangle$$

because the aggregation number increase $\left({\left(\frac{\partial {\left[N_{\mathrm{agg}}\right]}^{2}}{\partial \ln m_{2}}\right)}_{T}>0\right)$ is driven by surfactant-induced enhancement of surfactant–surfactant interaction (number correlation), $\Delta_{2}\left\langle \delta n_{2}^{l}\delta n_{2}^{l}\right\rangle >0$ . Thus, the existence of premicelles is the evidence for the cooperative nature of micelle formation. We emphasize that the increase of $N_{22}^{l}+1$ reduces the isotherm gradient, which is true regardless of the subcategories for the degree of surfactant self-association (premicelles and micelles).

Our conclusions above are not restricted to some surfactants (e.g., DTAB due to the availability of activity data below CMC) but is expected to apply to surfactants in general (e.g., SDS and Tween). First, CMC is a general feature of surfactants, at which surfactant self-assembly drives up $N_{22}^{l}+1$ , leading to the plateau (diminished gradient) of surface tension isotherms. The existence of CMC must be accompanied by the increase of $N_{22}^{l}+1$ , i.e., premicelles. Second, the linear region of the surface tension isotherm is another feature of surfactants in general, where surfactant adsorption is saturated. Consequently, “precipitous decline” must precede prior to saturation. Thus, our theory provides a direct link between these general characteristics of surfactants and the functional shape of the surface tension isotherms.

## Conclusion

Our goal was to understand the shape of a surface tension isotherm (i.e., how air/water surface tension changes with surfactant concentration) based on the surfactant interactions at the interface and in the bulk solution. Achieving this goal was made difficult by the unrealistic, oversimplistic assumptions in the classical models, such as the site-specific binding for surface adsorption and monodisperse micelles for surfactant self-aggregation in the bulk. Thus, the stoichiometric models (via the law of mass action) for adsorption and bulk self-association had to be replaced for interpretive clarity.

We have established a novel theory of surface tension isotherms founded on the statistical thermodynamic fluctuation theory. This was achieved by synthesizing our recent work on sorption isotherms and on the structures of complex solutions. We have established model-free interpretive tools for the gradient (first derivative) and curvature (second derivative) of a surface tension isotherm, in terms of a competition between surfactant interactions at the interface and in the bulk solution.

We have replaced the stoichiometric definition of the aggregation number (applicable only to monodisperse micelles) with the one based on surfactant number fluctuations, which can be evaluated from the surface tension gradient ratio between the linear and post-CMC regions. With fluctuation as its foundation, the general features of a surfactant surface tension isotherm (including the precipitous onset, linear region, CMC, and postmicellar region) have been explained mechanistically, without any need for model assumptions. Not only can the fluctuation theory capture polydisperse, non-stoichiometric associations but also the crucial importance of surfactant–surfactant exclusion for the surface coverage. Moreover, there is no longer a need to construct separate theories for surfactants and small molecules due to the universality of the fluctuation theory. A forthcoming paper will generalize our theory to co-molecule (e.g., surfactant and co-solvent) mixtures.

This paper has established a link between the functional shape of an experimental isotherm and the parameters of our statistical thermodynamic isotherm that capture the overall correlations and distributions of co-molecules at the interface. These parameters will serve as a clear target for microscopic elucidation (e.g., in terms of surfactant monolayer and electric double layers of ionic surfactants) via molecular simulations.

## Supplementary Material



## Notes

Debate on interpreting surface tension isotherms, activity-based formalism of the air/liquid interface, mole fraction representation of the Gibbs isotherm; second ln *m*_2_ derivative, electrolyte solution theory, simple sorption isotherms for air/liquid interfaces, relationship with the STAND model for aggregation number determination, determining the aggregation number from osmotic coefficient, expressing the excess aggregation number via the Kirkwood–Buff integrals, and relationship with Rosen and Kunjappu (PDF)

## References

[1] Rosen, M. J. ; Kunjappu, J. T. Surfactants and Interfacial Phenomena; Wiley: New York, 2012.

[2] Butt, H. ; Graf, K. ; Kappl, M. Physics and Chemistry of Interfaces; Wiley: New York, 2003.

[3] Adamson, A. W. ; Gast, A. P. Physical Chemistry of Surfaces, 6th ed.; Wiley: New York, 1997.

[4] Tanford, C. The Hydrophobic Effect: Formation of Micelles and Biological Membranes; Wiley: New York, 1973.

[5] Tanford C. (1974). Thermodynamics
of Micelle Formation: Prediction of Micelle Size and Size Distribution. Proc. Natl. Acad. Sci. U. S. A..

[6] Rusanov A. (1993). The Mass Action
Law Theory of Micellar Solutions. Adv. Coll.
Interface Sci..

[7] Rusanov A. I. (2017). On the
Problem of Determining Aggregation Numbers from Surface Tension Measurements. Langmuir.

[8] Shimizu S., Matubayasi N. (2024). Replacing the Langmuir Isotherm with
the Statistical
Thermodynamic Fluctuation Theory. J. Phys. Chem.
Lett..

[9] Shimizu S., Matubayasi N. (2025). Gas and Liquid Isotherms: The Need for A Common Foundation. Langmuir.

[10] Menger F. M., Shi L., Rizvi S. A. A. (2010). Additional Support
for a Revised Gibbs Analysis. Langmuir.

[11] Arkhipov V. P., Arkhipov R. V., Kuzina N. A., Filippov A. (2021). Study of the Premicellar
State in Aqueous Solutions of Sodium Dodecyl Sulfate by Nuclear Magnetic
Resonance Diffusion. Magn. Reson. Chem..

[12] Cui X., Mao S., Liu M., Yuan H., Du Y. (2008). Mechanism of Surfactant
Micelle Formation. Langmuir.

[13] LeBard D. N., Levine B. G., DeVane R., Shinoda W., Klein M. L. (2012). Premicelles
and Monomer Exchange in Aqueous Surfactant Solutions Above and Below
the Critical Micelle Concentration. Chem. Phys.
Lett..

[14] Bergström M. (1996). Derivation
of Size Distributions of Surfactant Micelles Taking into Account Shape,
Composition, and Chain Packing Density Fluctuations. J. Colloid Interface Sci..

[15] Basheva E. S., Kralchevsky P. A., Danov K. D., Ananthapadmanabhan K.
P., Lips A. (2007). The Colloid
Structural Forces as a Tool for Particle Characterization
and Control of Dispersion Stability. Phys. Chem.
Chem. Phys..

[16] Soper A., Castner E., Luzar A. (2003). Impact of
Urea on Water Structure:
A Clue to Its Properties as a Denaturant?. Biophys.
Chem..

[17] Sahle C. J., Schroer M. A., Juurinen I., Niskanen J. (2016). Influence of TMAO and
Urea on the Structure of Water Studied by Inelastic X-ray Scattering. Phys. Chem. Chem. Phys..

[18] Schöttl S., Lopian T., Prévost S., Touraud D., Grillo I., Diat O., Zemb T., Horinek D. (2019). Combined Molecular
Dynamics (MD) and Small Angle Scattering (SAS) Analysis of Organization
on a Nanometer-Scale in Ternary Solvent Solutions Containing a Hydrotrope. J. Colloid Interface Sci..

[19] Winkler R., Buchecker T., Hastreiter F., Touraud D., Kunz W. (2017). PPh_4_ Cl
in Aqueous Solution – the Aggregation Behavior of an Antagonistic
Salt. Phys. Chem. Chem. Phys..

[20] Dwivedi, D. ; Lepková, K. In Application and Characterization of Surfactants; Najjar, R. , Ed.; InTech: London, U.K., 2017.

[21] Manet S., Cuvier A.-S., Valotteau C., Fadda G. C., Perez J., Karakas E., Abel S., Baccile N. (2015). Structure of Bolaamphiphile
Sophorolipid Micelles Characterized with SAXS, SANS, and MD Simulations. J. Phys. Chem. B.

[22] Gapiński J., Szymański J., Wilk A., Kohlbrecher J., Patkowski A., Hołyst R. (2010). Size and Shape of Micelles Studied
by Means of SANS, PCS, and FCS. Langmuir.

[23] Zemb T., Diat O. (2010). What Can We Learn from
Combined SAXS and SANS Measurements of the
Same Sample Containing Surfactants?. J. Phys.
Conf. Ser..

[24] Patel A. D., Desai M. A. (2023). Progress in the
Field of Hydrotropy: Mechanism, Applications
and Green Concepts. Rev. Chem. Eng..

[25] Hodgdon T. K., Kaler E. W. (2007). Hydrotropic Solutions. Curr.
Opin. Colloid Interface Sci..

[26] Kunz W., Holmberg K., Zemb T. (2016). Hydrotropes. Curr. Opin. Colloid Interface Sci..

[27] Friberg S. E. (1997). Hydrotropes. Curr. Opin. Colloid Interface Sci..

[28] Bhatia A. B., Thornton D. E. (1970). Structural Aspects
of the Electrical Resistivity of
Binary Alloys. Phys. Rev. B.

[29] Nishikawa K. (1986). Simple Relationship
Between the Kirkwood–Buff Parameters and the Fluctuations in
the Particle Number and Concentration Obtained by Small-Angle X-ray
Scattering. Chem. Phys. Lett..

[30] Hayashi H., Nishikawa K., Iijima T. (1990). Easy Derivation of the Formula Relating
the Fluctuations of a Binary System to the X-ray scattering Intensity
Extrapolated to *s* = 0. J. Appl.
Crystallogr..

[31] Shimizu S., Matubayasi N. (2018). Statistical Thermodynamic Foundation for Mesoscale
Aggregation in Ternary Mixtures. Phys. Chem.
Chem. Phys..

[32] Kirkwood J.
G., Buff F. P. (1951). The Statistical
Mechanical Theory of Solutions. I. J. Chem.
Phys..

[33] Hall D. G. (1971). Kirkwood-Buff
Theory of Solutions. An Alternative Derivation of Part of It and Some
Applications. Trans. Faraday Soc..

[34] Ben-Naim A. (1977). Inversion
of the Kirkwood–Buff Theory of Solutions: Application to the
Water–Ethanol System. J. Chem. Phys..

[35] Shimizu S. (2004). Estimating
Hydration Changes upon Biomolecular Reactions from Osmotic Stress,
High Pressure, and Preferential Hydration Experiments. Proc. Natl. Acad. Sci. U. S. A..

[36] Ploetz E. A., Smith P. E. (2013). Local Fluctuations
in Solution: Theory and Applications. Adv. Chem.
Phys..

[37] Hall D. G. (1972). Thermodynamics
of Solutions of Interacting Aggregates by Methods Similar to Surface
Thermodynamics. Part 1.—General Equations for Multi-Component
Systems. J. Chem. Soc., Faraday Trans. 2.

[38] Hall D. G. (1974). Thermodynamics
of Solutions of Interacting Aggregates by Methods Similar to Surface
Thermodynamics. Part 2.—Solutions of Non-Associating Macromolecules. J. Chem. Soc., Faraday Trans. 2.

[39] Hall D. G. (1977). Thermodynamics
of Solutions of Interacting Aggregates by Methods Similar to Surface
Thermodynamics. Part 3.—Solutions of Ionic Surfactants in the
Absence and Presence of Electrolyte with a Common Counterion. J. Chem. Soc., Faraday Trans. 2.

[40] Shimizu S., Matubayasi N. (2021). Cooperativity
in Micellar Solubilization. Phys. Chem. Chem.
Phys..

[41] Garrido P.
F., Brocos P., Amigo A., Garcia-Rio L., Gracia-Fadrique J., Pineiro A. (2016). STAND: Surface Tension for Aggregation
Number Determination. Langmuir.

[42] Defay, R. ; Prigogine, I. ; Bellemans, A. Surface Tension and Adsorption; Longmans: London, U.K., 1966.

[43] Menger F. M., Shi L. (2009). Electrostatic Binding among Equilibrating 2-D and 3-D Self-Assemblies. J. Am. Chem. Soc..

[44] Menger F. M., Rizvi S. A. A. (2011). Relationship between Surface Tension and Surface Coverage. Langmuir.

[45] Mukherjee I., Moulik S. P., Rakshit A. K. (2013). Tensiometric Determination of Gibbs
Surface Excess and Micelle Point: A Critical Revisit. J. Colloid Interface Sci..

[46] Bermúdez-Salguero C., Gracia-Fadrique J. (2011). Analysis of
Gibbs Adsorption Equation and Thermodynamic
Relation between Gibbs Standard Energies of Adsorption and Micellization
through a Surface Equation of State. J. Colloid
Interface Sci..

[47] Laven J., De With G. (2011). Should the Gibbs Analysis Be Revised?. Langmuir.

[48] Szyszkowski B. V. (1908). Experimentelle
Studien über kapillare Eigenschaften der wässerigen
Lösungen von Fettsäuren. Z. Phys.
Chem..

[49] Langmuir I. (1917). The Constitution
and Fundamental Properties of Solids and Liquids: II. Liquids. J. Am. Chem. Soc..

[50] Frumkin A. (1925). Die Kapillarkurve
der höheren Fettsäuren und die Zustandsgleichung der
Oberflächenschicht. Z. Phys. Chem..

[51] Swenson H., Stadie N. P. (2019). Langmuir’s
Theory of Adsorption: A Centennial
Review. Langmuir.

[52] Peng M., Nguyen A. V. (2020). Adsorption of Ionic
Surfactants at the Air-Water Interface:
The Gap Between Theory and Experiment. Adv.
Coll. Interface Sci..

[53] Shimizu S., Matubayasi N. (2021). Sorption: A Statistical Thermodynamic Fluctuation Theory. Langmuir.

[54] Shimizu S., Matubayasi N. (2020). Fluctuation Adsorption Theory: Quantifying
Adsorbate-Adsorbate
Interaction and Interfacial Phase Transition from an Isotherm. Phys. Chem. Chem. Phys..

[55] Shimizu S., Matubayasi N. (2014). Preferential Solvation: Dividing Surface vs Excess
Numbers. J. Phys. Chem. B.

[56] Shimizu S., Matubayasi N. (2018). A Unified
Perspective on Preferential Solvation and
Adsorption Based on Inhomogeneous Solvation Theory. Phys. A.

[57] Frumkin A. N., Parsons R. (1964). A note on the paper “The description
of adsorption
at electrodes”. J. Electroanal. Chem..

[58] Shimizu S. (2020). Formulating
Rationally via Statistical Thermodynamics. Curr.
Opin. Colloid Interface Sci..

[59] Strey R., Viisanen Y., Aratono M., Kratohvil J. P., Yin Q., Friberg S. E. (1999). On the Necessity of Using Activities in the Gibbs Equation. J. Phys. Chem. B.

[60] Shimizu S., Matubayasi N. (2023). Understanding Sorption Mechanisms Directly from Isotherms. Langmuir.

[61] Shimizu S., Matubayasi N. (2023). Cooperativity in Sorption Isotherms. Langmuir.

[62] Shimizu S., Matubayasi N. (2023). Sorption from Solution: A Statistical
Thermodynamic
Fluctuation Theory. Langmuir.

[63] Shimizu S., Matubayasi N. (2022). Surface Area
Estimation: Replacing the BET Model with
the Statistical Thermodynamic Fluctuation Theory. Langmuir.

[64] Shimizu S., Matubayasi N. (2020). Intensive Nature of Fluctuations: Reconceptualizing
Kirkwood–Buff Theory via Elementary Algebra. J. Mol. Liq..

[65] Robinson, R. A. ; Stokes, R. H. Electrolyte Solutions, 2nd ed.; Butterworths: London, U.K., 1965.

[66] Lewis, G. N. ; Randall, M. ; Pitzer, K. S. ; Brewer, L. Thermodynamics, 2nd ed.; McGraw-Hill: New York, 1961.

[67] Gradštejn, I. S. ; Ryžik, J. M. ; Jeffrey, A. ; Zwillinger, D. ; Gradštejn, I. S. Table of Integrals, Series and Products, 7th ed.; Academic Press: Amsterdam, Netherlands, 2009.

[68] Kleinheins J., Shardt N., El Haber M., Ferronato C., Nozière B., Peter T., Marcolli C. (2023). Surface tension models
for binary aqueous solutions: a review and intercomparison. Phys. Chem. Chem. Phys..

[69] Phan C. M. (2021). The surface
tension and interfacial composition of water/ethanol mixture. J. Mol. Liq..

[70] Mahle J. J. (2002). An adsorption
equilibrium model for Type 5 isotherms. Carbon.

[71] La
Mesa C., Bonincontro A., Sesta B. (1993). Solution Properties of Octyl *β*-D Glucoside. Part 1: Aggregate Size. Shape and Hydration. Colloid Polym. Sci..

[72] López-Cervantes J. L., Sandoval-Ibarra F. D., Gracia-Fadrique J. (2021). Equilibrium Model for Estimating
Micellar Aggregation Number from Surface Equation. Fluid Phase Equilib..

[73] Prokhorov V. A., Rusanov A. (1990). Surface Tension and the Degree of Binding of Counterions
by Micelles in the Dodecyltrimethylammonium Bromide—Water System. Colloid J. USSR.

[74] Yamanaka M., Kaneshina S. (1990). Volumetric Behavior and Micelle Formation of Aqueous
Surfactant Mixtures. J. Solution Chem..

[75] De
Lisi R., Fisicaro E., Milioto S. (1988). Thermodynamic Properties and Conductivities
of Some Dodecylsurfactants in Water. J. Solution
Chem..

[76] Shimizu S., Matubayasi N. (2022). Ensemble Transformation
in the Fluctuation Theory. Phys. A.

[77] Lamch L., Leszczynska I., Dlugowska D., Szczesna Gorniak W., Batys P., Jarek E., Wilk K. A., Warszynski P. (2025). Synthesis
of New Cationic Dicephalic Surfactants and Their Nonequivalent Adsorption
at the Air/Solution Interface. Langmuir.

