# Supplementary material for: Surface Tension Isotherms: Reconceptualizing Adsorption, Self-Assembly, and Micelle Formation via the Fluctuation Theory
Source: Langmuir. 2026 Feb 9;42(7):5416–30. doi: 10.1021/acs.langmuir.5c05155 (PMC12937112; doi:10.1021/acs.langmuir.5c05155)
Supplement: Supplementary file 1 [file la5c05155_si_001.pdf]

Supporting Information
Surface Tension Isotherms: Reconceptualizing Adsorption, Self-Assembly, and Micelle Formation via the Fluctuation Theory

Seishi Shimizu*,† and Nobuyuki Matubayasi‡

†*York Structural Biology Laboratory, Department of Chemistry, University of York, Heslington, York YO10 5DD, United Kingdom*

‡*Division of Chemical Engineering, Graduate School of Engineering Science, Osaka University, Toyonaka, Osaka 560-8531, Japan*

E-mail: seishi.shimizu@york.ac.uk

Contents

A Debate on Interpreting Surface Tension Isotherms **S2**

B Activity-Based Formalism of the Air/Liquid Interface **S3**

C The Gibbs Isotherm along $\ln m_2$ **S7**

D The Second $\ln m_2$-Derivative **S10**

E Electrolyte Solution Theory **S12**

F Simple Sorption Isotherms for Air/Liquid Interfaces **S14**

G Relationship with the STAND Model for Aggregation Number Determination S16

H Determining the Aggregation Number from Osmotic Coefficient S17

I Expressing the Excess Aggregation Number via the Kirkwood-Buff Integrals S19

J Relationship with Rosen and Kunjappu S21

A Debate on Interpreting Surface Tension Isotherms

A.1 Onset of Surface Tension Decline

Surface tension isotherms exhibit a sudden onset of decline (**Figure 1**) when plotted against logarithmic concentrations. Menger et al. "wondered why the surface tension remain relatively constant in region A [in **Figure 1**] but then begin its precipitous decline only after saturation is ostensively reached at the beginning of region B."[10] This line of argument led them to an apparent paradox: "it seemed strange that the surface tension is affected by molecules populating the interface and yet surface tension decreases when molecules do not further adsorb",[10] which has led them to the controversial questioning the Gibbs adsorption isotherm itself.[10]

A.2 Near-Linear Decrease in Surface Tension

It has been observed that "[f]or concentrations below but near the CMC, the slope of the curve is essentially constant"[1] (see region B of **Figure 1**). How do surfactant molecules interact when surface tension decreases almost linearly? The first explanation, according to Rosen and Kunjappu, is that "[i]n this range, the interface is saturated with surfactant [. . .], and the continued reduction in the surface tension [. . .] is due mainly to an increased activity

[...] of the surfactant in the bulk phase rather than at the interface" (p 71 of Ref [1]). The second, according to Menger et al., is that "the surface tension decline in region B arises from a continuously increasing occupancy of the interface."[10] Thus, there are still contradictory explanations for this widely observed phenomenon, evidencing the lack of consensus on how surface tension isotherms should be interpreted mechanistically.

A.3 Critical Micelle Concentration

The surface tension suddenly levels off at CMC, i.e., "the concentration at which the monomeric form, in which the surfactant exists in very dilute solution, aggregates to form a surfactant cluster known as a micelle [...]."[1] Here, the key difference in view is whether, (a) "the surface concentration has reached a constant maximum value" below CMC, according to Rosen and Kunjappu,[1] or (b) the preference of surfactant molecules for "joining the micelles over entering an unsaturated interface", according to Menger et al.[10] According to (a), surface tension plateau is the direct consequence of micelle formation ("only the monomeric form contributes to the reduction of the surface or interfacial tension"[1]), whereas (b) considers that micelle formation only lowers the concentration at which surface tension start to plateau ("[i]f it were not for micelle formation, region B would continue its downward journey and then level off at the saturation point rather than at the critical micelle concentration (CMC)."[10]) These contradictory views necessitate a mechanistic clarification of why surface tension plateaus.

B Activity-Based Formalism of the Air/Liquid Interface

B.1 Gibbs Dividing surface

Here, we introduce the Gibbs dividing surface that enables us to focus on the surface excess of co-molecules.[55] Let $c_i^*(\vec{r})$ be the local concentration of species i, and c_i^a and c_i^l be the bulk

concentrations on the air and liquid sides. The overall interfacial effect is quantified in terms of the net excess of $c_2^*(\vec{r})$ from the reference bulk subsystems on the air (c_2^a) and liquid (c_2^l) sides, via[55]

$$\Gamma_{s2} = \frac{1}{\sigma}\int_{v^*} d\vec{r}\left[(c_2^*(\vec{r}) - c_2^\alpha) - \frac{c_2^l - c_2^a}{c_1^l - c_1^a}(c_1^*(\vec{r}) - c_1^\alpha)\right] \tag{B.1}$$

referred to as the surface excess, where c_i^α is defined as

$$c_i^\alpha \equiv \alpha c_i^l + (1-\alpha)c_i^a \tag{B.2}$$

with an arbitrary α.[55] We emphasize that the integration (eq B.1) converges for any α to a value independent of α. As is well-known, under this setup, positioning the Gibbs dividing surface appropriately can eliminate the solvent's surface excess, such that we can focus exclusively on the distribution of co-molecules in the vicinity of the defined surface.[54,55]

B.2 Setting up an Interface

Here, we derive the generalized Gibbs isotherm for air/liquid interfaces. This can be achieved through a straightforward extension of our previous results for solid/gas[54] and solid/liquid[62] interfaces with any interfacial geometry. We consider a mixture of solvent (species 1) and co-molecule (species 2) that forms an interface between the air and liquid phases. Analogously to solid/gas[54] and solid/liquid[62] interfaces, the grand canonical ensembles for the entire system ($*$) and for the air (a) and liquid (l) reference systems are

$$\Omega^* = -PV^* + \gamma_s\sigma, \qquad \Omega^l = -PV^l, \qquad \Omega^a = -PV^a \tag{B.3}$$

where γ_s is the surface tension, σ is the interfacial area, P is the pressure and V^*, V^l, and V^a are the volumes of the system, the liquid reference system, and the air reference system,

respectively. Under the volume conservation requirement,

$$V^* = V^l + V^a \tag{B.4}$$

along with the fact that the pressure P is common to all three systems, we obtain

$$\gamma_s \sigma = \Omega^* - \Omega^l - \Omega^a \tag{B.5}$$

B.3 Gibbs Dividing Condition

With this setup, now we introduce the Gibbs dividing condition algebraically, following our previous work.[54] Through the Legendre transformation, the thermodynamic function corresponding to the partially open ensemble, $Y(N_1, \mu_2, V, T)$ can be introduced via

$$Y^\tau = \Omega^\tau + \mu_1 N_1^\tau \tag{B.6}$$

for $\tau = *,\ l,\ a$. Combining eqs B.5 and B.6, we obtain[54]

$$\gamma_s \sigma = Y^* - Y^l - Y^a - \mu_1 \left(N_1^* - N_1^l - N_1^a\right) \tag{B.7}$$

In eq B.7, setting the surface excess of the solvent to zero, that is,

$$N_1^* - N_1^l - N_1^a = 0 \tag{B.8}$$

is equivalent to introducing the Gibbs dividing surface. Under eq B.8, eq B.7 simplifies to

$$\gamma_s \sigma = Y^* - Y^l - Y^a \tag{B.9}$$

Thus, surface tension has been linked to excess ("system minus references") thermodynamic functions for partially open ensembles. We emphasize that eq B.9 is mathematically identical

in form with our previous work for the solid/gas interface.[54]

B.4 The Gibbs Isotherm

Here, we derive the Gibbs isotherm by differentiating eq B.9 with respect to μ_2. This can be carried out straightforwardly via the mathematical analogy between eq B.9 and eq 14 of Ref [[54]] for air/liquid interface. Consequently, following the same mathematical procedure as eqs 15-19 of Ref [[54]], we obtain the following result.

$$-\sigma \left(\frac{\partial \gamma_s}{\partial \mu_2} \right)_T = \langle N_2^* \rangle - \left\langle N_2^l \right\rangle - \langle N_2^a \rangle \tag{B.10}$$

where $\langle N_2^* \rangle$,$\left\langle N_2^l \right\rangle$, and $\langle N_2^a \rangle$ are the number of cosolvents in the system, the liquid reference and the air reference, respectively. Using a well-known thermodynamic relationship, $d\mu_2 = RT \ln a_2$, eq B.10 can be rewritten as

$$-\frac{1}{RT} \left(\frac{\partial \gamma_s}{\partial \ln a_2} \right)_T = \frac{\langle N_2^* \rangle - \left\langle N_2^l \right\rangle - \langle N_2^a \rangle}{\sigma} \tag{B.11}$$

B.5 The Local Gibbs Isotherm per Area

Interfacial effect is confined within a finite distance,[53,54] which enables us to rewrite the right-hand side of eq B.11 as

$$-\frac{1}{RT} \left(\frac{\partial \gamma_s}{\partial \ln a_2} \right)_T = \frac{\langle n_2^* \rangle - \left\langle n_2^l \right\rangle - \langle n_2^a \rangle}{\sigma} \tag{B.12}$$

where $\langle n_2^* \rangle$,$\left\langle n_2^l \right\rangle$, and$\langle n_2^a \rangle$ are the ensemble-averaged cosolvent number in the local, bulk liquid, and bulk air subsystems, respectively. All the discussions in the main text will be based on these subsystems.

B.6 The Fluctuation Relationship

Evaluating the second $\ln a_2$-derivative of γ_s requires the following elementary statistical thermodynamic relationship (see eq 2 of Ref [3] and the paragraph below for further references):

$$\left(\frac{\partial \langle n_2^\tau \rangle}{\partial \ln a_2}\right)_T = \langle \delta n_2^\tau \delta n_2^\tau \rangle \tag{B.13}$$

where $\delta n_2^\tau = n_2^\tau - \langle n_2^\tau \rangle$ ($\tau = *,\ l,\ a$) is the deviation from the mean. Using eq B.13, we obtain the following.

$$-\frac{1}{RT}\left(\frac{\partial^2 \gamma_s}{\partial [\ln a_2]^2}\right)_T = \frac{\langle \delta n_2^* \delta n_2^* \rangle - \langle \delta n_2^l \delta n_2^l \rangle - \langle \delta n_2^a \delta n_2^a \rangle}{\sigma} \tag{B.14}$$

C The Gibbs Isotherm along $\ln m_2$

C.1 The Use of Bulk Solution Measurements for N_{22}^l

Here, we justify eq 11 in the main text, showing that the pressure change makes a negligible contribution.

Using $v_2' \simeq 300$ cm^3 mol^{-1} for DTAB[74] as an example, units conversion leads to $v_2' = 0.03$ kJ mol^{-1} bar^{-1}, showing that for $v_2'\left(\frac{\partial P'}{\partial m_2}\right)_{T;\text{interface}}$ to contribute to eq 11 an unrealistically large $\left(\frac{\partial P'}{\partial m_2}\right)_{T;\text{interface}}$ is required.

Thus, the term $v_2'\left(\frac{\partial P'}{\partial m_2}\right)_{T;\text{interface}}$ in eq 11 is negligible. This argument also holds in the development in Section D.

C.2 The First $\ln m_2$-Derivative

Here, we derive eq 12 in the main text, which plays a key role in interpreting surface tension isotherms plotted against co-molecule concentrations.

First, we focus on a two-component solution in the $\{T, P, n_1, \mu_2\}$ ensemble. carrying out

a μ_2-derivative of $\langle n_2 \rangle$ using the partition function, we obtain

$$\frac{1}{RT}\left(\frac{\partial \langle n_2 \rangle}{\partial \mu_2}\right)_{T,P,n_1} = \langle \delta n_2 \delta n_2 \rangle \tag{C.1}$$

Second, we carry out an ensemble transformation, $\{T, P, n_1, \mu_2\} \rightarrow \{T, v, n_1, \mu_2\}$, for the partial derivative in eq C.1. Starting with the general relationship,

$$\left(\frac{\partial \langle n_2 \rangle}{\partial \mu_2}\right)_{T,v,n_1} = \left(\frac{\partial \langle n_2 \rangle}{\partial \mu_2}\right)_{T,P,n_1} + \left(\frac{\partial \langle n_2 \rangle}{\partial P}\right)_{T,\mu_2,n_1} \left(\frac{\partial P}{\partial \mu_2}\right)_{T,v,n_1} \tag{C.2}$$

Using the chain rule,

$$\left(\frac{\partial \langle n_2 \rangle}{\partial P}\right)_{T,\mu_2,n_1} = -\left(\frac{\partial \mu_2}{\partial P}\right)_{T,n_1,n_2} \left(\frac{\partial \langle n_2 \rangle}{\partial \mu_2}\right)_{T,P,n_1} = -v_2 \left(\frac{\partial \langle n_2 \rangle}{\partial \mu_2}\right)_{T,P,n_1} \tag{C.3}$$

where v_2 is the partial molar volume of species 2 and was written as v_2' in eqs 10 and 11 and Section C.1. Using eq C.3, eq C.2 is rewritten as

$$\left(\frac{\partial \langle n_2 \rangle}{\partial \mu_2}\right)_{T,v,n_1} = \left(\frac{\partial \langle n_2 \rangle}{\partial \mu_2}\right)_{T,P,n_1} \left[1 - v_2 \left(\frac{\partial P}{\partial \mu_2}\right)_{T,v,n_1}\right] \tag{C.4}$$

As in Section C.1, using $v_2' = 0.03\,\mathrm{kJ\ mol^{-1}\ bar^{-1}}$ for DTAB[74] as an example, for $v_2 \left(\frac{\partial P}{\partial \mu_2}\right)_{T,v,n_1}$ to make a contribute to eq C.4, an unrealistically large $\left(\frac{\partial P}{\partial \mu_2}\right)_{T,v,n_1}$ is required. Thus, we neglect the second term of eq C.4, which simplifies to

$$\left(\frac{\partial \langle n_2 \rangle}{\partial \mu_2}\right)_{T,P,n_1} = \left(\frac{\partial \langle n_2 \rangle}{\partial \mu_2}\right)_{T,v_1=n_1/c_1,n_1} \tag{C.5}$$

where the subscript $T, v_1 = n_1/c_1, n_1$ emphasizes that v is constrained to be $v_1 = n_1/c_1$. Using the partition function for the $\{T, v, n_1, \mu_2\}$ ensemble with this constraint, we obtain

$$\frac{1}{RT}\left(\frac{\partial \langle n_2 \rangle}{\partial \mu_2}\right)_{T,v,n_1} = \langle \delta n_2 \delta n_2 \rangle \tag{C.6}$$

Combining all of the above, we obtain

$$\frac{1}{RT}\left(\frac{\partial\langle n_2\rangle}{\partial\mu_2}\right)_{T,P,n_1} = \langle\delta n_2 \delta n_2\rangle_{\{T,v,n_1,\mu_2\}} \tag{C.7}$$

where the subscript $\{T, v, n_1, \mu_2\}$ emphasizes the ensemble used to evaluate the fluctuation, which is the same as the reference system l for the interface.

Third, using eq 11 to incorporate the experimental setup for surface tension isotherm measurements, we can identify the bulk measurement $\left(\frac{\partial\langle n_2\rangle}{\partial\mu_2}\right)_{T,P,n_1}$ with the interfacial quantity $\left(\frac{\partial\langle n_2\rangle}{\partial\mu_2}\right)_{T,n_1}$, which leads straightforwardly to eq 12.

C.3 The Mole-Fraction Representation of the Gibbs Isotherm

We have adopted the cosolvent molality, m_2, because $\left(\frac{\partial \ln m_2}{\partial \ln a_2}\right)_T$ is directly related to the number of aggregation, $N^l_{22}+1$, in the bulk solution phase (eq 13 of the main text). However, the mole fraction of the cosolvent, x_2, is often used to plot the surface tension isotherms. Consequently, here we clarify how a $\ln x_2$-based isotherm can be related to the $\ln m_2$-based isotherm. To do so, we start with

$$\left(\frac{\partial \ln a_2}{\partial \ln x_2}\right)_T = \left(\frac{\partial \ln a_2}{\partial \ln m_2}\right)_T \left(\frac{\partial \ln m_2}{\partial \ln x_2}\right)_T \tag{C.8}$$

From the definitions of x_2 and m_2, they are interrelated via

$$x_2 = \frac{N^l_2}{N^l_1 + N^l_2} = \frac{\frac{N^l_2}{N^l_1}}{1 + \frac{N^l_2}{N^l_1}} = \frac{M_1 m_2}{1 + M_1 m_2} \tag{C.9}$$

where M_1 is the molecular weight of species 1. Differentiating eq C.9, we obtain

$$\left(\frac{\partial \ln x_2}{\partial \ln m_2}\right)_T = \frac{1}{1 + M_1 m_2} = \frac{N^l_1}{N^l_1 + N^l_2} = x_1 \tag{C.10}$$

where x_1 is the mole-fraction of the solvent. Combining eqs 13 of the main text and C.10 yields

$$-\frac{1}{RT}\left(\frac{\partial \gamma_s}{\partial \ln x_2}\right)_T = \frac{1}{x_1}\frac{\Gamma_{s2}}{1+N_{22}^l} \tag{C.11}$$

which is the Gibbs isotherm in the mole-fraction representation (eq 14 of the main text).

D The Second $\ln m_2$-Derivative

D.1 Exact Theory

Here, we derive eq 17 of the main text by converting the second $\ln m_2$ derivative to the $\ln a_2$-derivatives. Carrying out the $\ln m_2$-derivative of eq 16 of the main text yields

$$-\frac{1}{RT}\left(\frac{\partial^2 \gamma_s}{\partial\left[\ln m_2\right]^2}\right)_T = \frac{1}{\left(1+N_{22}^l\right)^2}\left[\left(\frac{\partial \Gamma_{s2}}{\partial \ln m_2}\right)_T\left(1+N_{22}^l\right) - \Gamma_{s2}\left(\frac{\partial N_{22}^l}{\partial \ln m_2}\right)_T\right] \tag{D.1}$$

Now we evaluate the two terms in [ ] of eq D.1. For the first term, we note that

$$\left(\frac{\partial \Gamma_{s2}}{\partial \ln m_2}\right)_T = \left(\frac{\partial \Gamma_{s2}}{\partial \ln a_2}\right)_T\left(\frac{\partial \ln a_2}{\partial \ln m_2}\right)_T \tag{D.2}$$

Using eq 7 of the main text and B.13, we obtain

$$\left(\frac{\partial \Gamma_{s2}}{\partial \ln a_2}\right)_T = \frac{\langle \delta n_2^* \delta n_2^* \rangle - \langle \delta n_2^l \delta n_2^l \rangle - \langle \delta n_2^a \delta n_2^a \rangle}{\sigma} \equiv \frac{\Delta_s \langle \delta n_2 \delta n_2 \rangle}{\sigma} \tag{D.3}$$

Combining eqs D.2, D.3, and 13 of the main text, we obtain

$$\left(\frac{\partial \Gamma_{s2}}{\partial \ln m_2}\right)_T\left(1+N_{22}^l\right) = \frac{\Delta_s \langle \delta n_2 \delta n_2 \rangle}{\sigma} \tag{D.4}$$

where $\Delta_s \langle \delta n_2 \delta n_2 \rangle$ signifies the enhancement of cosolvent fluctuation at the interface. For the second term in [ ] of Eq. D.1, with the help of eq 13 of the main text, we obtain the

following.

$$\left(\frac{\partial N_{22}^l}{\partial \ln m_2}\right)_T = \left(\frac{\partial N_{22}^l}{\partial \ln a_2}\right)_T \left(\frac{\partial \ln a_2}{\partial \ln m_2}\right)_T = \frac{1}{1+N_{22}^l}\left(\frac{\partial N_{22}^l}{\partial \ln a_2}\right)_T \tag{D.5}$$

With the help of the inhomogeneous solvation theory,

$$N_{22}^l = \left\langle n_2^l \right\rangle_2 - \left\langle n_2^l \right\rangle \tag{D.6}$$

where $\left\langle n_2^l \right\rangle_2$ is the mean number of cosolvent in the subsystem that contains a probe cosolvent fixed at the origin. Using eq D.6, $\left(\frac{\partial N_{22}^l}{\partial \ln a_2}\right)_T$ in eq D.5 evaluates to

$$\left(\frac{\partial N_{22}^l}{\partial \ln a_2}\right)_T = \left(\frac{\partial \left\langle n_2^l \right\rangle_2}{\partial \ln a_2}\right)_T - \left(\frac{\partial \left\langle n_2^l \right\rangle}{\partial \ln a_2}\right)_T = \left\langle \delta n_2^l \delta n_2^l \right\rangle_2 - \left\langle \delta n_2^l \delta n_2^l \right\rangle \equiv \Delta_2 \left\langle \delta n_2^l \delta n_2^l \right\rangle \tag{D.7}$$

with the help of eq B.13. We have introduced $\Delta_2 \left\langle \delta n_2^l \delta n_2^l \right\rangle$ as the enhancement of cosolvent fluctuation around a probe cosolvent. Substituting eq D.7 into eq D.5 yields

$$\left(\frac{\partial N_{22}^l}{\partial \ln m_2}\right)_T = \frac{\Delta_2 \left\langle \delta n_2^l \delta n_2^l \right\rangle}{1+N_{22}^l} \tag{D.8}$$

A straightforward combination of eqs D.1, D.4, and D.8 will lead to eq 17 of the main text. Introducing the aggregation number via $N_{\mathrm{agg}} = N_{22}^l + 1$ (see eq 33 of the main text), eq D.8 can also be expressed as

$$\left(\frac{\partial \left[N_{\mathrm{agg}}\right]^2}{\partial \ln m_2}\right)_T = 2\Delta_2 \left\langle \delta n_2^l \delta n_2^l \right\rangle \tag{D.9}$$

to clarify how $\Delta_2 \left\langle \delta n_2^l \delta n_2^l \right\rangle$ drives up the aggregation number, $N_{22}^l + 1$.

D.2 Incorporating Adsorption Saturation

Here, we consider a special case of eq 17 of the main text in which sorption has reached saturation. In this case, the curvature of a surface tension isotherm reflects the change of self-association in the bulk solution. We start with eq 16 of the main text with a constant

$\Gamma_{s2} \simeq \Gamma_{s2}^{sat}$, i.e.,

$$-\frac{1}{RT}\left(\frac{\partial \gamma_s}{\partial \ln m_2}\right)_T = \frac{\Gamma_{s2}^{sat}}{1+N_{22}^l} \tag{D.10}$$

whose differentiation with respect to $\ln m_2$ yields

$$-\frac{1}{RT}\left(\frac{\partial^2 \gamma_s}{\partial\left[\ln m_2\right]^2}\right)_T = \Gamma_{s2}^{sat}\left(\frac{\partial}{\partial \ln m_2}\frac{1}{N_{22}^l+1}\right)_T = -\frac{\Gamma_{s2}^{sat}}{\left(N_{22}^l+1\right)^2}\left(\frac{\partial N_{22}^l}{\partial \ln m_2}\right)_T \tag{D.11}$$

With the help of eq D.8, eq D.11 transforms to

$$\frac{1}{RT}\left(\frac{\partial^2 \gamma_s}{\partial\left[\ln m_2\right]^2}\right)_T = \Gamma_{s2}^{sat}\frac{\Delta_2\left\langle \delta n_2^l \delta n_2^l\right\rangle}{\left(1+N_{22}^l\right)^3} \tag{D.12}$$

Note that eq D.12 is a special case of eq 17 of the main text with the omission of the latter's first term. This is because $\left(\frac{\partial \Gamma_{s2}}{\partial \ln a_2}\right)_T = 0$ when $\Gamma_{s2} = \Gamma_{s2}^{sat}$, which leads to $\Delta_s\langle \delta n_2 \delta n_2\rangle = 0$ via eq 8 of the main text. We will apply eq D.12 as eq 52 of the main text to elucidate the surface tension isotherm around CMC.

E Electrolyte Solution Theory

Here, we generalize our theory to electrolyte solutions. To do so, consider a co-molecule (species 2) as salt composed of ν_+ cations and ν_- anions, with the total number of ions $\nu = \nu_+ + \nu_-$. (ν corresponds to the isotonic coefficient of the van't Hoff equation for osmotic pressure.) Following the classical thermodynamic approach to electrolyte solutions,[65,66] the mean activity ($a_\pm$) and molality ($m_\pm$) of ions can be introduced via

$$a_\pm = (a_2)^{\frac{1}{\nu}} \tag{E.1}$$

and the mean molality

$$m_\pm = \left(m_+^{\nu_+} m_-^{\nu_-}\right)^{\frac{1}{\nu}} = \left({\nu_+}^{\nu_+}{\nu_-}^{\nu_-}\right)^{\frac{1}{\nu}} m_2 \tag{E.2}$$

Note that $m_\pm = m_2$ for the special case of 1:1 salt. The standard state is taken such that[65]

$$\gamma_\pm = \frac{a_\pm}{m_\pm} \to 1 \qquad m_\pm \to 0 \tag{E.3}$$

With this preparation, the generalized Gibbs isotherm (eq 7) converts to

$$-\frac{1}{RT}\left(\frac{\partial \gamma_s}{\partial \ln a_\pm}\right)_T = \Gamma_{s\pm} = \nu \Gamma_{s2} \tag{E.4}$$

where $\Gamma_{s\pm}$ is defined as the surface excess of mean ions, related to the excess numbers of co-molecules as salt, Γ_{s2}.) To express the $\ln m_\pm$ gradient of the surface tension isotherm, we generalize the theory of bulk self-assembly (eq 13) to the electrolyte co-molecules via eq E.4, which yields

$$\left(\frac{\partial \ln a_\pm}{\partial \ln m_2}\right)_{T,P} = \left(\frac{\partial \ln a_\pm}{\partial \ln m_\pm}\right)_{T,P} = \frac{1}{N^l_{\pm\pm} + 1} \tag{E.5}$$

where the excess number of mean ions, $N^l_{\pm\pm}$, replaces N_{22}. Combining eqs E.4 and E.5, we obtain the expression for the surface tension isotherm gradient for electrolyte co-molecules, as a generalization of eq 16 of the main text, as

$$-\frac{1}{RT}\left(\frac{\partial \gamma_s}{\partial \ln a_\pm}\right)\left(\frac{\partial \ln a_\pm}{\partial \ln m_\pm}\right) = \frac{\Gamma_{s\pm}}{N^l_{\pm\pm} + 1} \tag{E.6}$$

Thus, the surface tension isotherm, plotted against salt molality (m_2) is interpreted as the competition between the sorption of mean ions ($\Gamma_{s\pm}$) and the bulk self-assembly of mean ions ($N^l_{\pm\pm} + 1$). Note that the surfactant aggregation number (eq 33 in the main text) can be generalized to electrolyte co-molecules as $N_{\text{agg}} = N^l_{\pm\pm} + 1$. We emphasize that $N^l_{\pm\pm} + 1$, just like $N^l_{22} + 1$, is, by definition, an excess number, whose relationship to the stoichiometric model[6,7] may provide further insight into micellization.

F Simple Sorption Isotherms for Air/Liquid Interfaces

Here, we derive two simple isotherm equations for air/liquid mixtures that are mathematically analogous to solid/vapor and solid/liquid interfaces.

F.1 The ABC Isotherm

Parallel to solid/vapor interfaces, we start with the combination of eqs 4, 8, and 9 of the main text,

$$\left(\frac{\partial}{\partial a_2} \frac{a_2}{\langle n_2^* \rangle - \langle n_2^l \rangle - \langle n_2^a \rangle}\right)_T = -\frac{\langle n_2^* \rangle N_{22}^* - \langle n_2^l \rangle N_{22}^l - \langle n_2^a \rangle N_{22}^a}{\left(\langle n_2^* \rangle - \langle n_2^l \rangle - \langle n_2^a \rangle\right)^2} \tag{F.1}$$

Equation F.1 for air/liquid has the same mathematical form as the solid/vapor counterpart (eq 3 of Ref [63]), and the same mathematical procedure (eqs 5-9 of Ref [63]) leads to the ABC isotherm for air/liquid interface, i.e.,

$$\Gamma_{s2} = \frac{a_2}{A_0 - B_0 a_2 - \frac{C_0}{2} a_2^2} \tag{F.2}$$

with the first two parameters (that will be used in the main text) expressed as

$$\frac{1}{A_0} = \left(\frac{\Gamma_{s2}}{a_2}\right)_{a_2 \to 0} = \frac{1}{\sigma}\left(\frac{\langle n_2^* \rangle - \langle n_2^l \rangle - \langle n_2^a \rangle}{a_2}\right)_{a_2 \to 0} \tag{F.3}$$

$$B_0 = -\left(\frac{\partial}{\partial a_2}\frac{a_2}{\Gamma_{s2}}\right)_{a_2 \to 0} = \sigma\left(\frac{\langle n_2^* \rangle N_{22} - \langle n_2^l \rangle N_{22}^l - \langle n_2^a \rangle N_{22}^a}{\left(\langle n_2^* \rangle - \langle n_2^l \rangle - \langle n_2^a \rangle\right)^2}\right)_{a_2 \to 0} \tag{F.4}$$

where we presented the two expressions for A_0 and B_0, the first to establish the analogy with the solid/gas interface (Cf. eq 9 of Ref [63]) and the second for the use in the main text. The subscript 0 emphasizes that the parameters are determined at the $a_2 \to 0$ limit. In practice, these parameters are determined by fitting the ABC isotherm (eq F.2) to an experimental isotherm measured over a finite concentration range of the co-molecule; hence,

the ABC isotherm should be considered an expansion around $a_2 = 0$, which is the reference point reached formally through extrapolation.[60,63] Note that eqs F.3 and F.4 differs from our previous paper in the appearance of σ, which comes from defining Γ_{s2} per surface area to be in conformity with experimental practice.

F.2 Polynomial Isotherm.

Here, we extend the cubic isotherm, derived for solid/liquid interfaces,[62] to air/liquid. To do so, let us start analogously to eq F3a of Ref [62]

$$\frac{\Gamma_{s2}}{a_2} = A' + B' a_2 + \frac{C'}{2} a_2^2 + \dots \tag{F.5}$$

where A', B', and C' are the parameters to be determined through a comparison of eq F.5 to eq F.2, which yields

$$A' = \frac{1}{A_0}, \quad B' = \frac{B_0}{A_0^2}, \quad C' = \frac{C_0}{A_0^2} + \frac{2B_0^2}{A_0^3} \tag{F.6}$$

Rewriting eq F.5 using eq F.6 yields

$$\Gamma_{s2} = \frac{1}{A_0} a_2 + \frac{B_0}{A_0^2} a_2^2 + \frac{1}{2}\left(\frac{C_0}{A_0^2} + \frac{2B_0^2}{A_0^3}\right) a_2^3 + \dots \tag{F.7}$$

We emphasize that both the ABC and cubic isotherms share the same parameters, A_0, B_0, and C_0.

F.3 Surface Tension Isotherms Derived from the ABC Sorption Isotherm

In Table 1 of the main text, for $B_0^2 > -2A_0C_0$, the arctanh isotherm is valid within $-\sqrt{2A_0C_0 + B_0^2} < C_0 a_2 + B_0 < \sqrt{2A_0C_0 + B_0^2}$. A more general isotherm equation is the

following:

$$\frac{\gamma_s - \gamma_s^o}{RT} = \frac{1}{\sqrt{2A_0C_0 + B_0^2}} \left[\ln \left| \frac{1 - \frac{C_0 a_2 + B_0}{\sqrt{2A_0C_0+B_0^2}}}{1 + \frac{C_0 a_2 + B_0}{\sqrt{2A_0C_0+B_0^2}}} \right| - \ln \left| \frac{1 - \frac{B_0}{\sqrt{2A_0C_0+B_0^2}}}{1 + \frac{B_0}{\sqrt{2A_0C_0+B_0^2}}} \right| \right] \tag{F.8}$$

This equation reduces to the AB expression of eq 25 at the limit of $C_0 \to 0$.

G Relationship with the STAND Model for Aggregation Number Determination

The STAND model by Garrido et al combines the Szyszkowski-Langmuir isotherm with a simple stoichiometric model for micelle formation for non-electrolyte surfactants.[41] Here, we replace the stoichiometric foundation of this model with the fluctuation theory. Following the McMillan-Mayer theory, the molarity c_2 can be expanded as a series of a_2. Incorporating only the a_2 and a_2^m terms, the expansion simplifies to

$$c_2 \simeq a_2 + \lambda_m a_2^m \tag{G.1}$$

representing the dominance of surfactant monomer and m-body correlation in the solution. This is analogous to the stoichiometric binding model adopted by Garrido et al.,[41]

$$c_2 = c_F + mK_m c_F^m \tag{G.2}$$

in which m "free surfactant" molecules, with the concentration c_F, form a micelle with the stoichiometric association constant, K_m. Clearly, there is a mathematical analogy between the fluctuation theory (eq G.1) and the stoichiometric STAND model (eq G.2). Note, in the STAND model, that "both the adsorption isotherm and the Gibbs adsorption equation are [...] expressed in terms of the free surfactant concentration."[41] This corresponds to adopting the a_2-based AB adsorption isotherm in combination with our eq G.1. Consequently, the

"free surfactant concentration" of the STAND model corresponds to the surfactant activity a_2 of our statistical thermodynamic fluctuation theory. Following this analogy to the STAND model, we can combine the a_2-based Langmuir isotherm and eq G.1 to evaluate the $\ln c_2$-gradient of the surface tension isotherm, which yields

$$\left(\frac{\partial \gamma_s}{\partial \ln c_2}\right)_T = \left(\frac{\partial \gamma_s}{\partial \ln a_2}\right)_T \left(\frac{\partial \ln a_2}{\partial \ln c_2}\right)_T = -RTn_m \frac{K_L a_2}{1+K_L a_2} \frac{a_2 + \lambda_m a_2^m}{a_2 + m\lambda_m a_2^m} \tag{G.3}$$

The gradient (eq G.3) reduces from $-RTn_m$ at $a_2 \simeq 0$ to $-RTn_m/m$ at large a_2. This reduction of gradient captures the plateau above CMC in the surface tension isotherm. Thus, the STAND model has now been founded on the fluctuation theory.

H Determining the Aggregation Number from Osmotic Coefficient

Here, we link the aggregation number with the osmotic coefficient.

H.1 Nonelectrolyte Solutions

The osmotic coefficient, ϕ, is linked to the solvent activity, a_1, via

$$\ln a_1 = -M_1 m_2 \phi \tag{H.1}$$

where (M_1 is the molecular weight (kg/mol) of the solvent.[66] This necessitates a relationship between a_1 and $N_{22}^l + 1$. To do so, using the Gibbs-Duhem equation under constant pressure,[66]

$$d\ln a_2 + \frac{1}{M_1 m_2} d\ln a_1 = 0 \tag{H.2}$$

Using eq H.2, eqs 12 and 13 of the main text transforms to

$$\frac{1}{N_{22}^{l}+1} = -\frac{1}{M_1 m_2}\left(\frac{\partial \ln a_1}{\partial \ln m_2}\right)_{T,P} = -\frac{1}{M_1}\left(\frac{\partial \ln a_1}{\partial m_2}\right)_{T,P} \tag{H.3}$$

Combining eqs H.1 and H.3, we obtain

$$\frac{1}{N_{22}^{l}+1} = \left(\frac{\partial m_2 \phi}{\partial m_2}\right)_{T,P} \tag{H.4}$$

H.2 Electrolyte Solutions

The osmotic coefficient, ϕ, is linked to a_1 via[65,66]

$$\ln a_1 = -\nu M_1 m_2 \phi \tag{H.5}$$

Since the aggregation number for electrolytes is defined as $N_{\pm\pm}+1$, we need to link $\ln a_1$ to $\ln a_{\pm}$.[65] To do so, the first step is to rewrite the isothermal-isobaric Gibbs-Duhem equation (eq H.2) in terms of the mean ion activity, $a_{\pm}$, to[65]

$$d\ln a_{\pm} + \frac{1}{\nu M_1 m_2} d\ln a_1 = 0 \tag{H.6}$$

Using eq H.6, the excess number relationship (eq 13) becomes

$$\frac{1}{N_{\pm\pm}+1} = -\frac{1}{\nu M_1 m_2}\left(\frac{\partial \ln a_1}{\partial \ln m_{\pm}}\right) \tag{H.7}$$

Using eq H.5, eq H.7 simplifies through eq E.2 to

$$\frac{1}{N_{\pm\pm}+1} = -\frac{1}{\nu M_1}\left(\frac{\partial \ln a_1}{\partial m_2}\right) \tag{H.8}$$

Combining eqs H.7 and H.8 yields

$$\frac{1}{N_{\pm\pm}+1} = \left(\frac{\partial m_2 \phi}{\partial m_2}\right) \tag{H.9}$$

Thus, the aggregation number for electrolytes ($N_{\pm\pm}+1$, eq H.9) and nonelectrolytes ($N_{22}^{l}+1$, eq H.4) can be evaluated in the same way.

I Expressing the Excess Aggregation Number via the Kirkwood-Buff Integrals

Here, we express N_{22}, defined in the partially open ensemble $\{T, P, n_1, \mu_2\}$, in terms of the Kirkwood-Buff integrals[32,34] defined in the $\{T, v, \mu_1, \mu_2\}$ ensemble. This can be achieved by the statistical variable transformation.[64,76] Since the co-molecule/solvent mole ratio and its variance is invariant under ensemble transformation $\{T, P, n_1, \mu_2\} \to \{T, v, \mu_1, \mu_2\}$

$$\frac{\langle n_2 \rangle + (\delta n_2)_{\{\mu_1\}}}{\langle n_1 \rangle + (\delta n_1)_{\{\mu_1\}}} = \frac{\langle n_2 \rangle + (\delta n_2)_{\{n_1\}}}{\langle n_1 \rangle} \tag{I.1}$$

where the subscripts $\{\mu_1\}$ and $\{n_1\}$ are the shorthand expressions for $\{T, v, \mu_1, \mu_2\}$ and $\{T, P, n_1, \mu_2\}$, respectively. The Maclaurin expansion of eq I.1 yields

$$(\delta n_2)_{\{n_1\}} = (\delta n_2)_{\{\mu_1\}} - \frac{\langle n_2 \rangle}{\langle n_1 \rangle} (\delta n_1)_{\{\mu_1\}} \tag{I.2}$$

This leads to

$$\left\langle (\delta n_2)_{\{n_1\}} (\delta n_2)_{\{n_1\}} \right\rangle = \left\langle (\delta n_2)_{\{\mu_1\}} (\delta n_2)_{\{\mu_1\}} \right\rangle \tag{I.3}$$

$$-2\frac{\langle n_2 \rangle}{\langle n_1 \rangle} \left\langle (\delta n_2)_{\{\mu_1\}} (\delta n_1)_{\{\mu_1\}} \right\rangle + \left(\frac{\langle n_2 \rangle}{\langle n_1 \rangle}\right)^2 \left\langle (\delta n_1)_{\{\mu_1\}} (\delta n_1)_{\{\mu_1\}} \right\rangle \tag{I.4}$$

Using the definition of the Kirkwood-Buff integral,[55]

$$G_{ij} = v\frac{\left\langle (\delta n_i)_{\{\mu_1\}} (\delta n_j)_{\{\mu_1\}} \right\rangle - \delta_{ij} \langle n_i \rangle}{\langle n_i \rangle \langle n_j \rangle} \tag{I.5}$$

Equation I.4 converts to

$$\frac{\left\langle (\delta n_2)_{\{n_1\}} (\delta n_2)_{\{n_1\}} \right\rangle}{\langle n_2 \rangle} = c_2 (G_{22} - 2G_{21} + G_{11}) + \frac{1}{x_1} \tag{I.6}$$

where c_2 is the molarity of species 2. Using the definition of N_{22}, we obtain

$$x_1 (N_{22} + 1) = c_2 x_1 (G_{22} - 2G_{21} + G_{11}) + 1 \tag{I.7}$$

Combining eq I.7 with eq 14 of the main text, we recover the well-known result from the Kirkwood-Buff theory[32,34]

$$\left(\frac{\partial \ln a_2}{\partial \ln x_2}\right)_{T,P} = \frac{1}{c_2 x_1 (G_{22} - 2G_{21} + G_{11}) + 1} \tag{I.8}$$

which validates our statistical variable transformation.

Note that the large positive $x_1 (N_{22} + 1)$ underlying the plateau in the plot of $\ln a_2$ against $\ln x_2$ at large x_2 confirms that a large $N_{22} + 1$ is responsible for the plateau. From the Kirkwood-Buff perspective (i.e., the right-hand side of eq I.8), $(G_{22} - 2G_{21} + G_{11})$ is responsible for a large net self-association.

The excess number as the measure of co-molecule self-assembly is the direct consequence of the Gibbs dividing condition (eq 2 of the main text) for defining an interface. This enables us to focus on the surface-co-molecule and co-molecule-co-molecule interactions that are mediated by the surrounding solvent molecules. Our co-molecule-centric approach in the $\{T, v, n_1, \mu_2\}$ ensemble contrasts with the conventional Kirkwood-Buff theory in the grand

canonical ($\{T, v, \mu_1, \mu_2\}$) ensemble,[34]

$$\left(\frac{\partial \ln a_2}{\partial \ln c_2}\right)_{T,P} = \frac{1}{1 + c_2 \left(G_{22} - G_{12}\right)} \tag{I.9}$$

which employs molarity ($c_2 = n_2/v$) as the concentration scale, signifying the preferential co-molecule-co-molecule association (G_{22}) compared to co-molecule-solvent (G_{21}). The explicit appearance of co-molecule-solvent interaction in the Kirkwood-Buff theory is the consequence of adopting the grand canonical subsystem which allows both solvent and co-molecule to come into and out of it. However, these two approaches to capturing self-assembly (N_{22}^l vs $c_2 \left(G_{22} - G_{21}\right)$) are mutually interconvertible via ensemble transformation.[64,76] Nevertheless, our choice of working with N_{22}^l, while delegating the solvent as the mediating medium for co-molecule interactions, comes from the required consistency with the adsorption theory.

J Relationship with Rosen and Kunjappu

Here, we show that our theory agrees with the explanation of the linear region by Rosen and Kunjappu (p 71 of Ref [1]). Let us start with rewriting eq 7 of the main text as a relationship between the increments of surface tension ($\delta\gamma_s$) and of $\ln a_2$ ($\delta \ln a_2$) as

$$\delta\gamma_s = -RT\Gamma_{s2}\ \delta \ln a_2 \simeq -RT\Gamma_{s2}\ \delta \ln c_2 \tag{J.1}$$

In this framework, when "the interface is saturated with surfactant"[1] Γ_{s2} is a constant, "the continued reduction in the surface tension"[1] (i.e., a negative $\delta\gamma_s$ in eq J.1) "is due mainly to an increased activity [...]] of the surfactant in the bulk phase rather than at the interface"[1] (i.e., $\delta \ln a_2 > 0$ in eq J.1).
